# Pathogenomic analyses of *Shigella* isolates inform factors limiting shigellosis prevention and control across LMICs

**DOI:** 10.1038/s41564-021-01054-z

**Published:** 2022-01-31

**Authors:** Rebecca J. Bengtsson, Adam J. Simpkin, Caisey V. Pulford, Ross Low, David A. Rasko, Daniel J. Rigden, Neil Hall, Eileen M. Barry, Sharon M. Tennant, Kate S. Baker

**Affiliations:** 1 Clinical Infection, Microbiology and Immunity, Institute of Infection, Veterinary and Ecological Sciences, The University of Liverpool, Liverpool, UK; 2 Biochemistry and Systems Biology, Institute of Systems, Molecular and Systems Biology, The University of Liverpool, Liverpool, UK; 3 Gastrointestinal Infections and Food Safety (One Health), United Kingdom Health Security Agency, London, UK; 4 Earlham Institute, Norwich Research Park, Norwich, UK; 5 Department of Microbiology and Immunology, Institute for Genome Sciences, University of Maryland School of Medicine, Baltimore, MD USA; 6 School of Biological Sciences, University of East Anglia, Norwich, UK; 7 Center for Vaccine Development and Global Health, University of Maryland School of Medicine, Baltimore, MD USA

**Keywords:** Bacterial genomics, Conjugate vaccines

## Abstract

*Shigella* spp. are the leading bacterial cause of severe childhood diarrhoea in low- and middle-income countries (LMICs), are increasingly antimicrobial resistant and have no widely available licenced vaccine. We performed genomic analyses of 1,246 systematically collected shigellae sampled from seven countries in sub-Saharan Africa and South Asia as part of the Global Enteric Multicenter Study (GEMS) between 2007 and 2011, to inform control and identify factors that could limit the effectiveness of current approaches. Through contemporaneous comparison among major subgroups, we found that *S. sonnei* contributes ≥6-fold more disease than other *Shigella* species relative to its genomic diversity, and highlight existing diversity and adaptative capacity among *S. flexneri* that may generate vaccine escape variants in <6 months. Furthermore, we show convergent evolution of resistance against ciprofloxacin, the current WHO-recommended antimicrobial for the treatment of shigellosis, among *Shigella* isolates. This demonstrates the urgent need to integrate existing genomic diversity into vaccine and treatment plans for *Shigella*, providing a framework for the focused application of comparative genomics to guide vaccine development, and the optimization of control and prevention strategies for other pathogens relevant to public health policy considerations.

## Abstract (web summary)

Genomic analyses of 1,246 *Shigella* isolates collected from seven LMICs reveal the important role of *Shigella* genomic diversity in vaccination design and *Shigella* evolution of resistance against currently recommended antibiotic treatments, underscoring the use of pathogenomics for shigellosis prevention and control.

## Main

Shigellosis is a diarrhoeal disease responsible for approximately 212,000 annual deaths and accounting for 13.2% of all diarrhoeal deaths globally^1^. The Global Enteric Multicenter Study (GEMS) was a large case-control study conducted between 2007 and 2011, investigating the aetiology and burden of moderate-to-severe diarrhoea (MSD) in children <5 years old in low- and middle-income countries (LMICs)^2^. GEMS revealed shigellosis as the leading bacterial cause of diarrhoeal illness in children, who represent a major target group for vaccination^3^. The aetiological agents are *Shigella*, a Gram-negative genus comprising *S. flexneri*, *S. sonnei*, *S. boydii* and *S. dysenteriae*, with the former two species causing the majority (90%) of attributable shigellosis in children in LMICs^3^. Currently, the disease is primarily managed through supportive care and antimicrobial therapy. However, there has been an increase in antimicrobial resistance (AMR) among *Shigella*^4^. Particularly concerning is the rise in resistance against the fluoroquinolone antimicrobial ciprofloxacin, the current World Health Organization (WHO)-recommended treatment, such that fluoroquinolone-resistant (FQR) *Shigella* is one of a dozen pathogens for which WHO notes new antimicrobial therapies are urgently needed^5^. The disease burden and increasing AMR of *Shigella* call for improvements in treatment and management options for shigellosis, and substantial momentum has built to rise to this challenge.

However, there is no licenced vaccine widely available for *Shigella* and one of the main challenges in its development is the considerable genomic and phenotypic diversity of the organisms^6^. The distinct lipopolysaccharide O-antigen structures of *Shigella* determine its serotype and is responsible for conferring the short- to medium-term serotype-specific immunity following infection^7–10^. Hence, considerable efforts are focused on generating O-antigen-specific vaccines. However, except for *S. sonnei* that has a single serotype, all species encompass multiple diverse serotypes: 14 serotypes/subserotypes for *S. flexneri*, 19 for *S. boydii* and 15 for *S. dysenteriae*^11^. Thus, for serotype-targeted vaccine approaches, multivalent vaccines are proposed to provide broad protection against the disease^12^. While O-antigen conjugates are a leading strategy, challenge studies have recently demonstrated poor clinical efficacy^13,14^. An attractive alternative and/or complement to serotype-targeted vaccine formulations are specific-subunit vaccines that target highly conserved proteins and may offer broad protection. There are several candidates in development that have demonstrated protection in animal models^15,16^, but the degree of antigenic variation in these targets among the global *Shigella* population remains unknown. Other strategies being explored include vaccines combining protein and serotype antigens, such as Generalized Modules of Membrane Antigens (GMMA), which involves use of outer membrane particles derived from genetically modified *S. sonnei* to elicit a stronger immune response^17^. However, GMMA also failed to demonstrate clinical efficacy against shigellosis in a recent challenge study^18^, indicating the continuing challenges of *Shigella* vaccinology.

Whole-genome sequencing analysis (WGSA) provides sufficient discriminatory power to resolve phylogenetic relationships and characterize the diversity of bacterial pathogens, which are essential to informing vaccine development and other aspects of disease control^19,20^. However, these critical analysis tools are yet to be applied to a pathogen collection appropriate for broadly informing shigellosis control in the critical demographic of children in LMICs. Here we apply WGSA to *Shigella* isolates sampled during GEMS, representing 1,246 systematically collected isolates from across seven nations in sub-Saharan Africa and South Asia with some of the highest childhood mortality rates^2,21^. We found evidence of the potential benefit of genomic subtype-based targeting, characterized pathogen features that will complicate current vaccine approaches, and highlighted regional differences in *Shigella* diversity, as well as determinants of AMR, including convergent evolution towards resistance against currently recommended treatments. Our analysis of this unparalleled pathogen collection informs the control and prevention of shigellosis in those populations most vulnerable to the disease.

## Regional diversity of *Shigella* spp. across LMICs

**Fig. 1:**
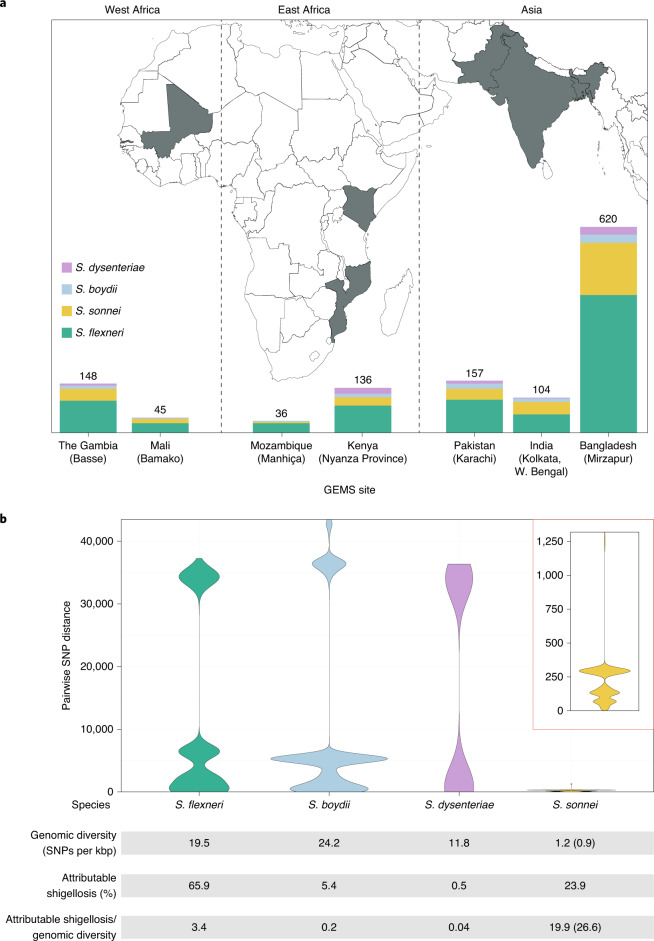
The diversity of *Shigella* spp. across seven LMICs. **a**, Stacked bar graphs illustrate the number of isolates from each *Shigella* spp. sequenced from GEMS and used in the current study, grouped by country. The seven countries from GEMS are highlighted in grey on the map and the selected field site(s) from each country are shown in brackets. **b**, Violin plots of pairwise genomic distances (in SNPs) among *Shigella* isolates within subgroups. Inset: a magnified plot for *S. sonnei*. The table below the plots shows the genomic diversity (as measured by the total number of SNPs per kbp (Methods)), the contribution to GEMS shigellosis burden and the shigellosis burden relative to genomic diversity for each species. For *S. sonnei*, the genomic diversity and shigellosis burden relative to genomic diversity calculated excluding the two outliers are shown in brackets. Source data

**Extended Data Fig. 1:**
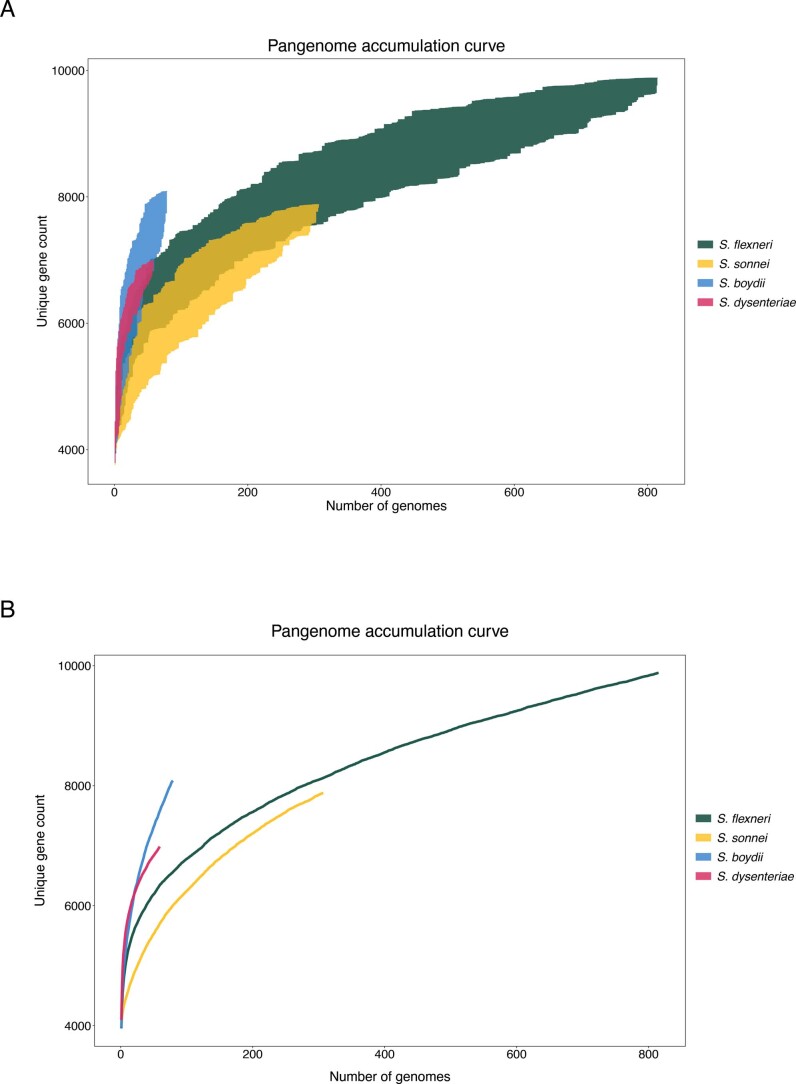
Pangenome accumulation curve of *S. flexneri*, *S. sonnei*, *S. boydii* and *S. dysenteriae*. Each curve demonstrates the number of unique protein coding genes in the pangenome as a new genome is randomly added. Random permutation of the data were subsampled 100 times, in which genomes are subsampled without replacement at each iteration. The x-axis display the number of genomes and the y-axis shows the minimum and maximum range of unique gene count after each iteration in (A) and the mean value in (B). Source data

**Extended Data Fig. 2:**
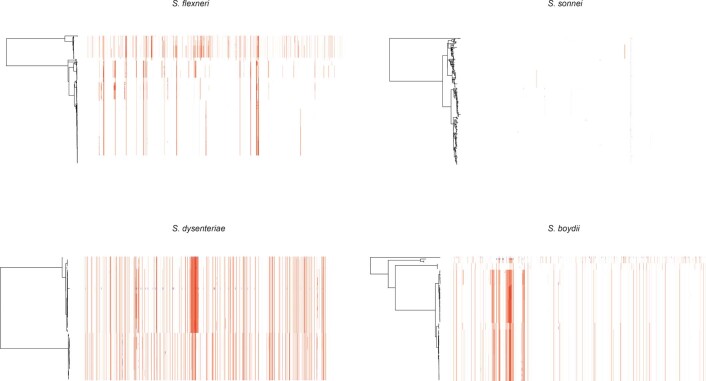
Detection of recombination among *Shigella* genomes. ML phylogenetic tree of each species are displayed on the left. Coloured columns represents regions of putative recombination with elevated SNP density detected across the genome. Columns highlighted in red represents recombination detected in multiple isolates and blue represents recombination detected in a single isolate. Source data

To date, this is the largest representative dataset of *Shigella* genomes from LMICs (*n* = 1,246), collected across seven sites from Asia, West Africa and East Africa, comprising 806 *S. flexneri*, 305 *S. sonnei*, 75 *S. boydii* and 60 *S. dysenteriae* (Fig. 1a). To compare the genomic diversity of *Shigella* species, we determined the distributions of pairwise single-nucleotide polymorphism (SNP) distances and scaled the total detected SNPs against the length of the chromosome (in kbp) for each species (Fig. 1b). This revealed that *S. boydii* contained the greatest diversity (24.2 SNPs per kbp), followed by *S. flexneri* (19.5 SNPs per kbp) and *S. dysenteriae* (11.8 SNPs per kbp), with *S. sonnei* being >9.8-fold less diverse (1.2 SNPs per kbp) or >13.1-fold less diverse (0.9 SNPs per kbp) when excluding two outliers (see below, Fig. 1b). Thus, *S. sonnei* caused more disease relative to genomic diversity than *S. flexneri* (5.9-fold), *S. dysenteriae* (497.5-fold) or *S. boydii* (99.5-fold) (Fig. 1b). However, when stratified by serotype/subserotype or genomic subtype, *S. sonnei* had a more comparable diversity and less pronounced increase in disease burden relative to genomic diversity (1.1–22.1-fold higher by serotype/subserotype and 1.2–4.9-fold higher by genomic subtype) (Supplementary Figs. 1 and 2). Further analyses revealed that the reduced diversity of *S. sonnei* (measured in chromosomal SNPs) was also reflected by a reduced accessory genome repertoire (Extended Data Fig. 1) and less recombination events across the genomes (Extended Data Fig. 2) relative to other species. This indicates the value of vaccination against *S. sonnei* as a comparatively conserved target relative to disease burden, and its comparability to subtypes of other *Shigella* spp.

**Fig. 2:**
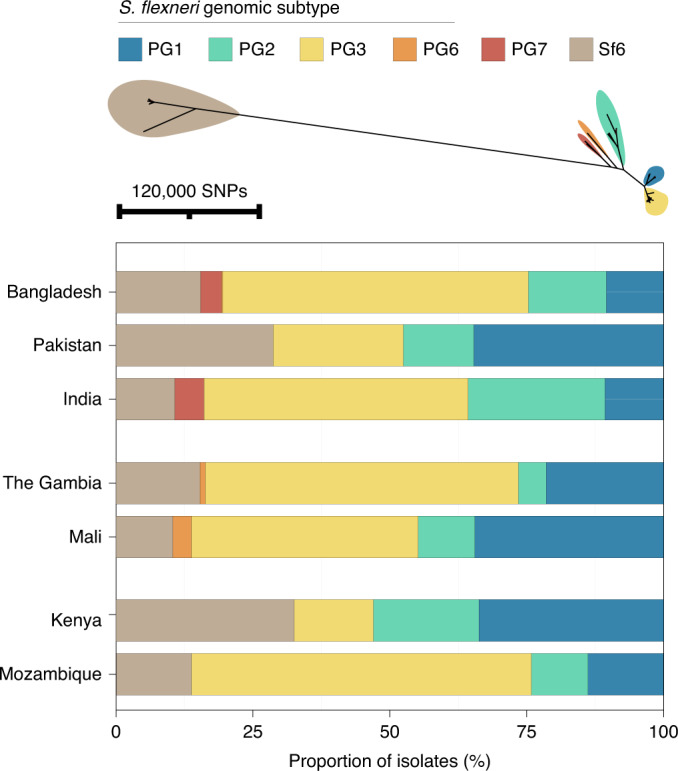
The diversity of *S. flexneri* genomic subtypes across seven GEMS study sites. Top: an unrooted maximum likelihood phylogenetic tree of *S. flexneri* genomes identified six distinct genomic subtypes, each highlighted in a different colour according to the key displayed above the tree. Bottom: barplot showing the relative frequencies of the subtypes at each GEMS site.

**Extended Data Fig. 3:**
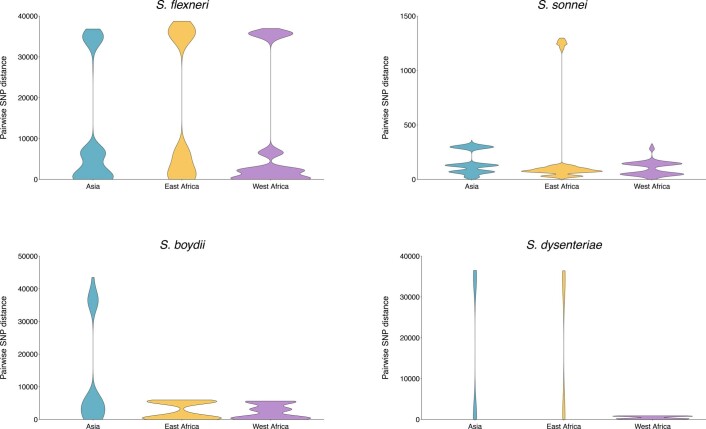
Regional genomic diversity of GEMS *Shigella*. Comparison of regional genomic diversity of *Shigella* spp, as measured by pairwise core SNP distance (x-axis) across GEMS study sites (Asia: Bangladesh, India and Pakistan; East Africa: Kenya and Mozambique; West Africa: The Gambia and Mali) for *S. flexneri*, *S. sonnei*, *S. boydii* and *S. dysenteriae*. Source data

**Extended Data Fig. 4:**
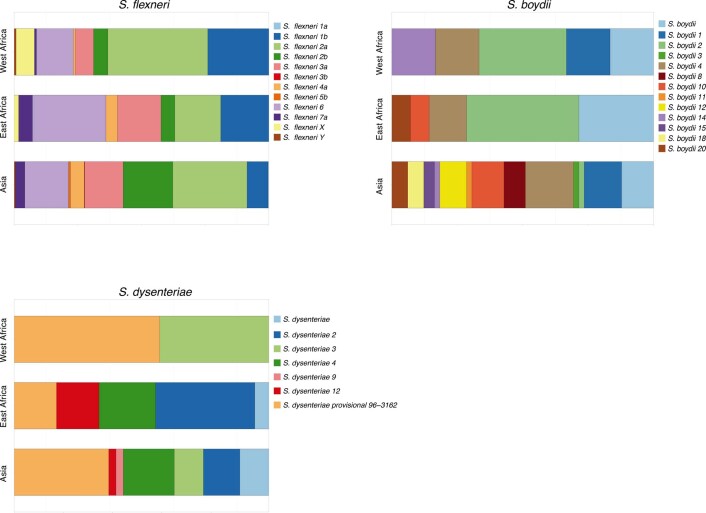
Regional serotypes/subserotypes diversity of GEMS *Shigella*. Regional serotypes/subserotypes diversity of *Shigella* spp across GEMS study sites for *S. flexneri*, *S. boydii* and *S. dysenteriae*. Each barplot represents the relative frequencies of serotypes/subserotypes at each region according to the inlaid key. Source data

Early global population structure studies revealed that each *Shigella* species is delineated into multiple WGSA subtypes^22–25^. Specifically, *S. flexneri* comprises seven phylogroups (PGs)^22^ and *S. sonnei*, five lineages^26^. To describe the genomic epidemiology of the GEMS *Shigella* within existing frameworks, we constructed species phylogenetic trees and integrated these with epidemiological metadata and publicly available genomes. The *S. flexneri* phylogeny revealed two distinct lineages separated by ~34,000 SNPs; one comprising five previously described PGs^22^ and a distant clade comprising largely *S. flexneri* serotype 6 isolates (herein termed Sf6), contributing distinctly to the disease burden of each country (Fig. 2 and Supplementary Fig. 3). Phylogenetic analysis of *S. sonnei* revealed that all but two isolates belonged to the globally dominant multidrug resistant (MDR) Lineage III^23^ (Supplementary Fig. 4). For *S. boydii* and *S. dysenteriae*, a total of three and two previously described phylogenetic clades^25,27^ were identified, respectively (Supplementary Fig. 5). Marked phylogenetic association of isolates with country of origin prompted an examination of species genomic diversity by region (East Africa, West Africa and Asia) and revealed that while *S. flexneri* diversity was comparable across regions, diversity varied by region for the remaining species (Extended Data Fig. 3). Specifically, *S. sonnei* was more genomically diverse in East Africa owing to the presence of two Lineage II isolates from Mozambique. For *S. boydii*, Asia contained greater diversity than African regions, owing to isolates belonging to additional clades. *S. dysenteriae* diversity was lower in West Africa relative to other regions by virtue of having only one circulating clade. Except for *S. sonnei*, similar trends were also observed for regional *Shigella* serotype/subserotype diversity (Extended Data Fig. 4). These geographical differences highlight the importance of considering regional variations during vaccine development and that vaccine candidates should be evaluated across multiple regions.

One limitation of the GEMS dataset is its constraints in geographical regions and time (being sampled between 2007 and 2011). However, several pieces of evidence support the utility of GEMS as being representative of *Shigella* in time and space. Specifically, the prevalence and regional distribution of *Shigella* serotypes across African GEMS sites were similar to those observed in the replicate Vaccine Impact of Diarrhea in Africa (VIDA) study conducted between 2015 and 2018^28^. Furthermore, recent large-scale genomic analyses of *S. sonnei* revealed that isolates sampled from a broad range of South Asian countries belonged to the same genomic subtype as the majority of GEMS isolates^26^. Finally, publicly available data for *S. flexneri* from LMICs sampled until 2021 were phylogenetically admixed with GEMS isolates (see below and Extended Data Fig. 7). Thus, GEMS has ongoing relevance as being representative of the diversity of *Shigella* targeted for control.

## Genomic subgroups as an alternative targeting method

As GEMS was a case-control study, the dataset comprised *Shigella* case isolates derived from patients with MSD and control isolates from children without diarrhoea^2^ (see Methods). To explore the utility of vaccination targeting genomic subtype (relative to targeting serotype) for *S. flexneri*, we determined the relative effect size of the dominant subtype on the epidemiological outcome of shigellosis (that is, isolates derived from case patients rather than from controls). The dominant genomic subtype was PG3, which comprised the majority (47%, 378/806) of total isolates, as well as case (50%, 341/687) isolates, with some regional variation (Fig. 2). This resulted in an increased odds ratio ofcase status (OR = 2.3, 95% CI = 1.5–3.6, *P* = 0.0001) for PG3 compared with other genomic subtypes (PGs and Sf6) (Methods and Supplementary Table 4). The association of cases with the dominant serotype, *S. flexneri* serotype 2a (accounting for 29% (234/806) of total isolates and 31% (210/687) of case isolates) also resulted in an increased odds ratio of case status (OR = 1.9, 95% CI = 1.7–3.2, *P* = 0.0099) (Supplementary Table 4). However, the higher prevalence of cases and larger effect size on case status of PG3 relative to serotype 2a offer compelling evidence that targeting vaccination by phylogroup might offer broader coverage per licenced vaccine relative to a serotype-specific approach. Hence, finding common surface-exposed antigens that are conserved within phylogroups causing the major burden of disease may be an effective vaccine design approach that can provide greater efficacy than serotype-targeted vaccines.

## Diversity of *S. flexneri* relevant to serotype-targeted vaccines

**Fig. 3:**
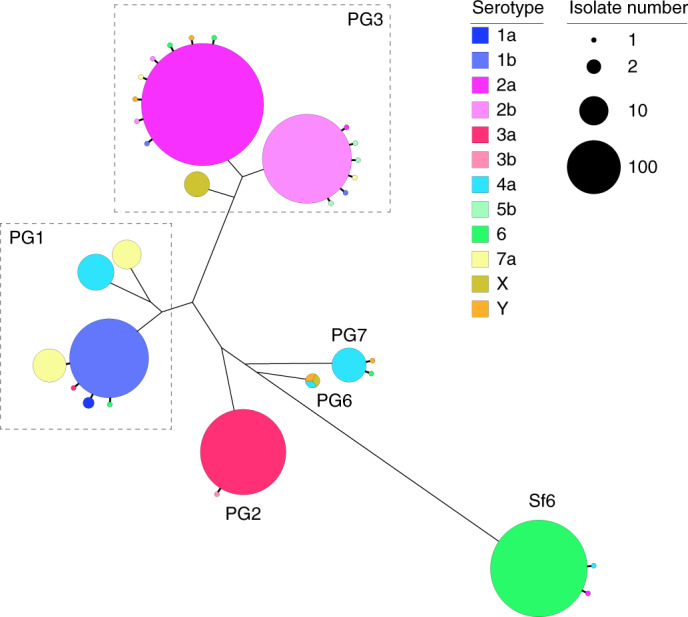
Diversity of *S. flexneri* population with respect to serotype switching. The unrooted *S. flexneri* phylogenetic tree is shown with the five phylogroups (PG1–PG7) and Sf6 labelled accordingly. For each genomic subtype, monophyletic clusters of the dominant serotype are shown collapsed into bubbles coloured according to the inlaid key. Single isolate or groups of isolates within a subtype of an alternative serotype are represented by further branches, indicating a single serotype switch. The dashed rectangles group together multiple serotypic clusters belonging to the same PG. Bubble size indicates the number of isolates within a single cluster.

**Extended Data Fig. 5:**
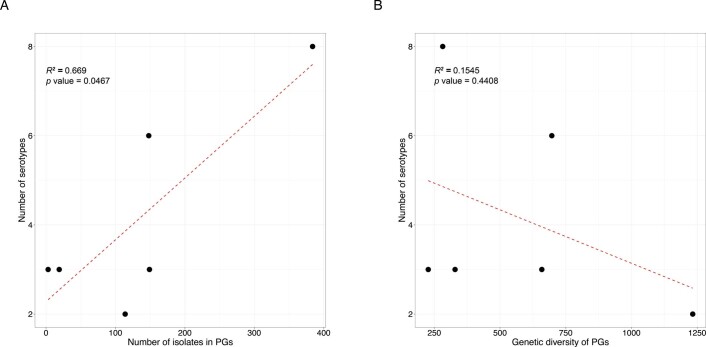
Association of *S. flexneri* serotype diversity with different properties of a genomic subtype. Each of the six subtypes identified among *S. flexneri* (PG1-PG7 and Sf6), the number of different serotypes is displayed along the y-axis and plotted against (A) the number of isolates within the subtype and (B) the genetic diversity of the subtype, as measured by pairwise core SNP distance and plotted along the x-axis. Linear regression analysis was performed to assess the association between serotype diversity and the different properties of subtypes. The regression coefficient of determination (R^2^) and *p*-value are displayed on the top left of each plot. Source data

The development of serotype-targeted vaccines is complicated by the diversity and distribution of serotypes, which are heterogenous over time and geography^8,21,29,30^. Furthermore, genetic determinants of O-antigen modification are often encoded on mobile genetic elements^31,32^ that can move horizontally among and within bacterial populations, causing the recognized, but poorly quantified phenomenon of serotype switching^22,30,31^, and resulting in the rapid escape of infection-induced immunity against homologous serotypes. For our analyses of serotype switching, we focused on *S. flexneri* owing to its high disease burden and serotypic diversity. Phenotypic serotyping data were overlaid onto the phylogeny and revealed that while there was a generally strong association of genotype (that is, PG/Sf6) with serotype (Fisher’s exact test, *P* < 2.20 × 10^−16^), multiple serotypes were observed for each genotype (Fig. 3). The greatest serotype diversity was observed in PG3, comprising seven distinct serotypes and two subserotypes. Correlation of serotypic diversity (number of serotypes) and genomic diversity (maximum pairwise SNP distance within genotype) revealed no evidence for an association (Extended Data Fig. 5). However, a significant positive correlation of serotypic diversity with the number of isolates in each genotype was found, indicating that serotype diversity scales with prevalence.

**Extended Data Fig. 6:**
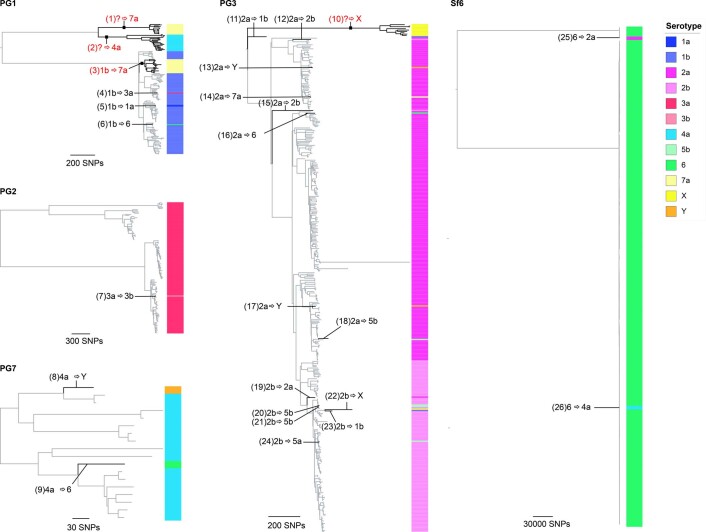
Serotype switching events across *S. flexneri* genomic subtypes. ML phylogenetic tree of each subtype was generated based on core genome SNPs. Serotypes determined through biochemical serotyping are displayed on the right-hand side of each tree, and coloured according to the inlaid key. The 26 inferred serotype switching events occurring along the phylogenetic branches are labelled accordingly. Numbers inside each backets represents switch IDs, with further details provided in Supplementary Table 4. Where the dominant serotype cannot be determined, a question mark is displayed, indicating switch from unknown ancestral type. Serotype switching events resulting in more than two descendant isolates are highlighted in red.

To qualitatively and quantitatively determine serotype switching across *S. flexneri*, we examined the number of switches occurring within each genotype. A switching event was inferred when a serotype emerged (either as a singleton or monophyletic clade) that was distinct from the majority (>65%) serotype within a genotype (Fig. 3 and Extended Data Fig. 6). PG6 was excluded from the analysis, as only three isolates from GEMS belonged to this genotype and a dominant serotype could not be inferred. Quantitatively, this revealed that serotype switching was infrequent, with only 26 independent switches (3.3% of isolates) identified across the five *S. flexneri* genotypes. Although the frequency of switching varied across the genotypes, statistical support for an association of serotype switching with genotype fell short of significance (Fisher’s exact test, *P* = 0.09). Qualitatively, the majority (22/26) of switching resulted in a change of serotype, with few (4/26) resulting in a change of subserotype. Examination of O-antigen modification genes revealed that serotype switching was facilitated by changes in the presence or absence of various phage-encoded *gtr* and *oac* genes in the genomes, as well as point mutations in these genes (Supplementary Table 5). Our data also revealed that few (4/26) switching events resulted in more than two descendant isolates (Extended Data Fig. 6). This indicates that while natural immunity drives the fixation of relatively few serotype-switched variants in the short term, the potential pool of variants that could be driven to fixation by vaccine-induced selective pressure following a serotype-targeted vaccination programme is much larger.

To estimate the probable timeframe over which serotype switching events might be expected to occur, we estimated the divergence time of the phylogenetic branch giving rise to each switching event. To streamline the analysis, we focused on two subclades of PG3, the most prevalent phylogroup, in which seven independent serotype switching events were detected (Supplementary Fig. 6). On the basis of the timeframes observed within our sample (spanning 4 years from 2007 to 2010), serotype switching was estimated to occur within an average of 348 d, ranging from 159 d (95% highest posterior density (HPD): 16–344) to 10,206 d (28 years) (95% HPD: 5,494–15,408) (Supplementary Table 6). Taken together, our data show that although serotype-switching frequency is low, it can occur over relatively short timeframes and lead to serotype replacement such that non-vaccine serotypes could replace vaccine serotypes following a vaccination programme, as has been observed for *Streptococcus pneumoniae*^33,34^. Consequently, serotype switching may impact the long-term effectiveness of vaccines that only provide serotype-specific protection against O-antigens. This highlights the advantages of protein-based or multivalent component approaches, such as the Invaplex or live attenuated vaccines that target both carbohydrates and protein antigens^6,35^.

## Heterogeneity among *Shigella* vaccine protein antigens

Although conserved antigen-targeted vaccines can overcome some hurdles of serotype-targeted vaccines, they are also subject to complications arising from genetic diversity. Hence, we performed detailed examination of six protein antigens that are currently in development and have demonstrated protection in animal models (Supplementary Table 1). First, we assessed the distribution of the candidates among GEMS *Shigella* isolates, which revealed that the proportional presence of antigens varied across species and with genetic context (Supplementary Fig. 7a). Specifically, genes encoded on the virulence plasmid (*ipaB*, *ipaC*, *ipaD*, *icsP*) were present in >85% of genomes for each species, with the exception of *S. sonnei*. The low proportion (≤5%) of virulence plasmid encoded genes detected among *S. sonnei* was caused by a similarly low detection of the virulence plasmid among *S. sonnei* (6%) (Supplementary Fig. 7b), which probably arose due to loss during sub-culture^36^. In contrast, the chromosomally encoded *ompA* was present in >98% of all isolates. While the *sigA* gene (carried on the chromosomally integrated SHI-1 pathogenicity island) was present in 99% of *S. sonnei* genomes, it was identified in only 63% of *S. flexneri* genomes. Notably, among *S. flexneri* genomes, the *sigA* gene was exclusively found in PG3 and Sf6, and was present in >96% of isolates in each genotype (Supplementary Fig. 3), indicating an appropriate distribution for targeting the two genotypes. Second, we assessed the antigens for amino acid variation and modelled the probable impact of detected variants, since antigen variation may also lead to vaccine escape, as demonstrated for the P1 variant of SARS-CoV-2^37,38^. We determined the distribution of pairwise amino acid (aa) sequence identities per antigen against *S. flexneri* vaccine strains for each species (see Methods). Overall, sequence identities were >90%, but varied with antigen (Supplementary Fig. 7a). For example, OmpA was present in the highest proportion of genomes, but showed ~5% sequence divergence, while SigA was present in fewer genomes, but exhibited little divergence (<0.5%) among species. The least conserved sequence was IpaD, ranging from 3 to 7% divergence within species.

**Fig. 4:**
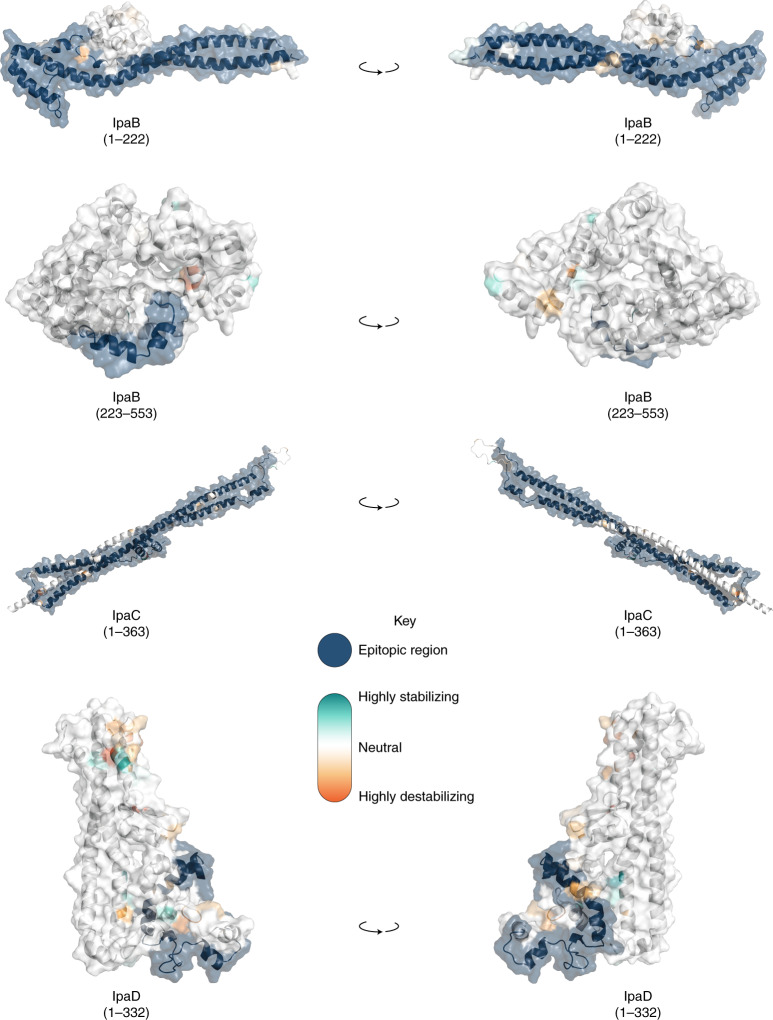
Visualization of mutations and its predicted effect on modelled IpaB, IpaC and IpaD protein antigens. Visualization of mutations on modelled proteins IpaB, IpaC and IpaD. The protein residue ranges modelled are shown in brackets. Blue regions represent empirically determined epitopes. Mutations identified within the proteins are coloured using the scale shown in the inlaid key. Visualisations in the right hand column are 180-degree rotations of the models relative to the left hand column.

**Extended Data Fig. 7:**
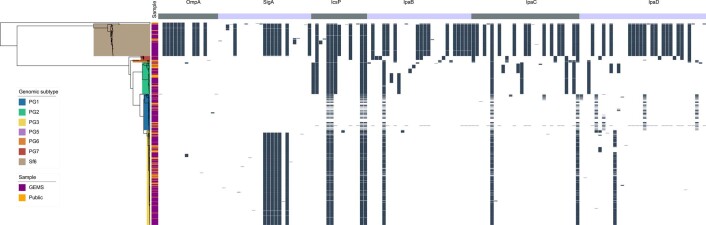
Vaccine antigen variation among *S. flexneri* subtypes. ML phylogenetic tree of 806*S. flexneri* GEMS and 236 publicly available genomes, based on core genome SNPs is displayed on the left. The six subtypes identified among the population are highlighted in different colours according to the inlaid key. The alternating grey and purple colour blocks displayed above the top panel represents the six antigen vaccine candidates assessed in the current study. The matrix in the center demonstrates presence (in black) of aa variation for each antigen vaccine. Only variable sites are displayed. The full list of variable sites represented are available in Supplementary Table 8.

Not all antigenic variation will affect antibody binding, so we performed in silico analyses of the detected variants to assess whether they may compromise the antigens as vaccine targets. Again, we focused our analyses on *S. flexneri* owing to its high disease burden and the probable complication of serotype-based vaccination strategies for this species. Furthermore, as *Shigella* vaccines are likely to be used broadly across LMICs, we expanded the analyses to include an additional 236 publicly available *S. flexneri* genomes (collected from 2007 and 2021, and sampled from various countries across Asia and Africa), which were phylogenetically admixed with the GEMS isolates (Extended Data Fig. 7). A total of 148 aa variants were detected across the six antigens, 58 (39%) of which were associated with genotype (that is, belonging to either PGs 1–5 or Sf6). Among the total variants detected, only 15 (10%) were unique to the publicly available genomes (Extended Data Fig. 7 and Supplementary Table 8), indicating that the GEMS dataset captured the majority of the diversity across LMICs. We then determined if aa variants were located in immunogenic regions (that is, epitope/peptide fragment) (Supplementary Fig. 8) and assessed their potential destabilization of protein structure through in silico protein modelling. For IpaB, IpaC and IpaD, the epitopes have been empirically determined^39,40^. The sequence and location of peptide fragments of SigA, IcsP and OmpA used in vaccine development are available^41,42^. Variants located within the immunogenic regions were identified for all antigens relative to PG3 reference sequences (Methods and Fig. 4). Only 5 of 148 variants were predicted to be highly destabilizing to protein structure, and these occurred in: OmpA (residue 89) at a periplasmic turn, SigA (residues 1233 and 1271) in adjacent extracellular turns in the translocator domain (Supplementary Fig. 9), IcsP (residue 191) within the extracellular region of the beta barrel, and IpaD (residue 247) within a beta-turn-beta motif flanking the intramolecular coiled-coil (Fig. 4). None of the five destabilizing variants were located within the epitope/peptide region of the vaccine candidates.

While it remains possible that mutations could affect antigenicity through the disruption of folding or global stability, it is less likely than if they occurred in immunogenic regions. Our results thus indicate that it is less likely that existing natural variation will compromise antigen-based vaccine candidates for *Shigella* compared with serotype-based vaccines. However, any in silico approaches have limitations and functional immunological experiments will be required to determine the true impact of these variations on the antigen structure and its antigenicity. Furthermore, the knowledge base regarding the structure of antigens is currently incomplete. For example, there was no suitable template available for IpaC, and some epitopes were predicted to be in membrane regions which should be inaccessible to antibodies, indicating the need for more accurate publicly available protein structures to be developed for many of the vaccine antigen candidates. Finally, 90% of the antigenic variants were captured by GEMS, further supporting the representativeness of this dataset across time and space. Nevertheless, the presence of an additional destabilizing mutation in the more recent publicly available data highlights the need for ongoing surveillance across LMICs.

## Region-specific details of antimicrobial resistance

**Fig. 5:**
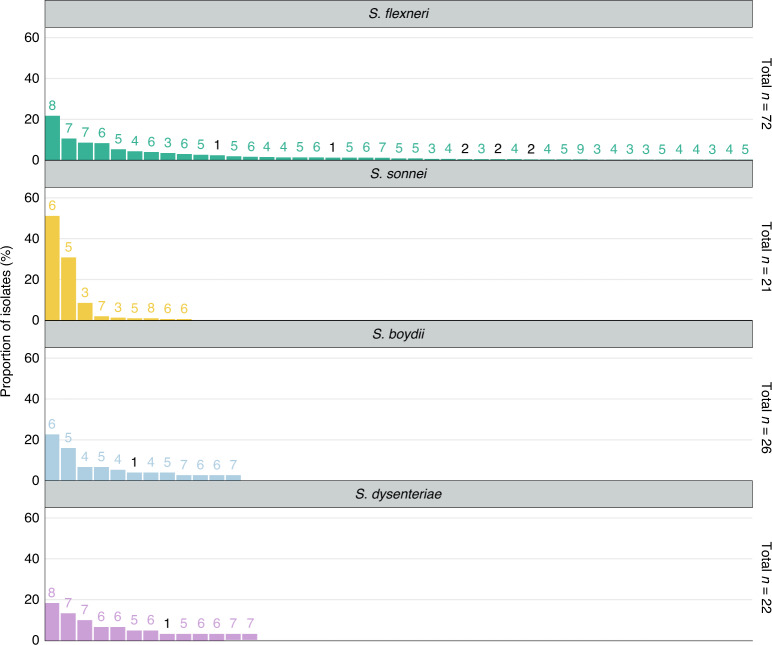
AMR genotypic profile diversity among *Shigella* spp. Each barplot represents the frequencies of AMR genotypic profiles among individual *Shigella* species. The number of bars shown along the *x* axis represents the number of unique genotypic AMR profiles detected in each species and plotted against the proportion of isolates belonging to each profile (*y* axis). The numbers above each profile indicate the number of antimicrobial classes impacted by the genotype. Profiles that impact only one or two antimicrobial classes (that is, are not MDR) are highlighted in black. AMR profiles identified in only a single isolate were not plotted and are displayed in Extended Data Fig. 9. The total number of profiles detected within each species are displayed on the right-hand side of each plot. Source data

**Extended Data Fig. 8:**
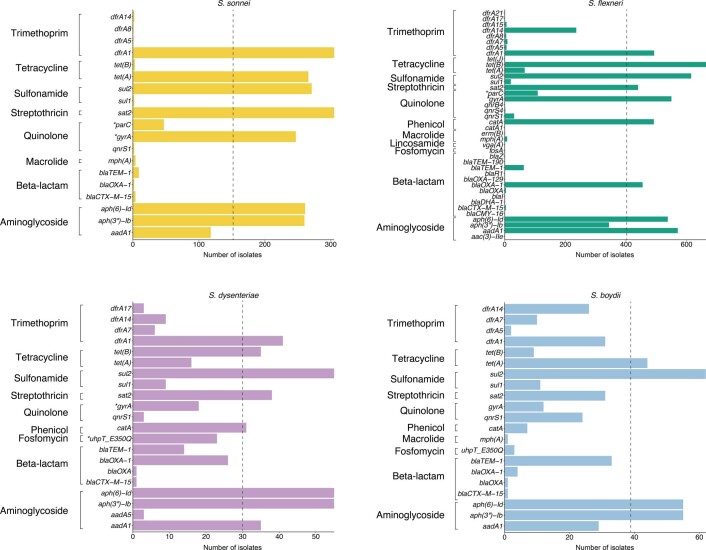
Prevalence of genetic determinants conferring AMR among *Shigella* spp. Barplots shows the number of genetic determinants detected in *S. sonnei, S. flexneri*, *S. dysenteriae* and *S. boydii* isolates that confer resistance or reduced susceptibility to various antimicrobials. Genes and point mutations (indicated with an asterisk) are plotted along the y-axis and grouped by drug class (displayed on the left). The dashed lines highlight genetic determinants identified in half or more of the isolates for each species. Source data

**Extended Data Fig. 9:**
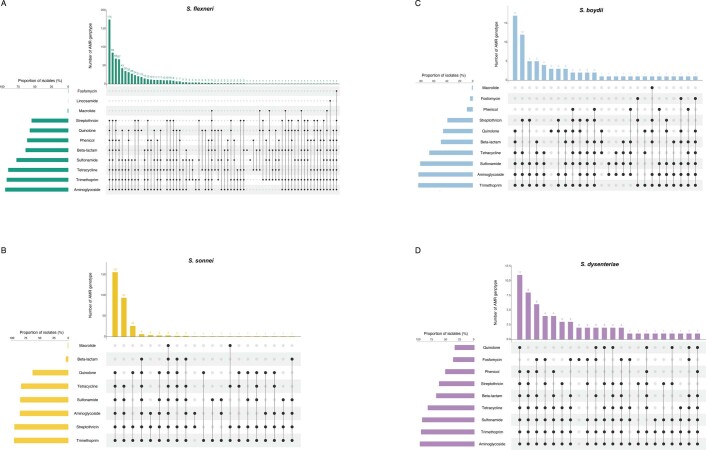
Diversity of AMR genotype resistance profiles. UpSet plots illustrate the AMR genotype resistance profiles for (A) *S. flexneri*, (B) *S. sonnei*, (C) *S. boydii* and (D) *S. dysenteriae*. Genotypic AMR profiles are shown in the combination matrix in the center panel. Each column represents a unique genotypic profile, where each black dot represents presence of a genetic determinant conferring resistance or reduced susceptibility to a drug class (displayed on the left). The vertical the barplot above the matrix displays the number of isolates with a particular profile, with the exact number of isolates displayed above each bar. The horizontal barplot on the left of the matrix illustrates the proportion of isolates containing AMR genetic determinants associated with a drug class. Source data

Until a licenced vaccine is available, we must continue to treat shigellosis with supportive care and antimicrobials, for which the current WHO recommendation is the fluoroquinolone, ciprofloxacin^43^. However, FQR *Shigella* is currently on the rise and spreading globally^44^. To examine AMR prevalence among GEMS isolates for evaluating treatment recommendations, we screened for known genetic determinants (horizontally acquired genes and point mutations) conferring resistance or reduced susceptibility to antimicrobials. Although we used only minimal phenotypic data, phenotypic resistance and genotypic prediction correlate well in *S. flexneri* and *S. sonnei*^45,46^. Our analysis revealed that 95% (1,189/1,246) of isolates were multidrug resistant (MDR), carrying AMR determinants against three or more antimicrobial classes (Fig. 5). *S. flexneri* exhibited the greatest diversity of AMR determinants, with a total of 45 identified determinants across the population, comprising 38 AMR genes and 7 point mutations (Extended Data Fig. 8 and Supplementary Table 2), and an extensive AMR genotype diversity of 72 unique resistance profiles (Fig. 5 and Extended Data Fig. 9). In contrast, *S. sonnei* exhibited the least diversity, with only 23 AMR determinants and 21 unique resistance profiles. An intermediate and comparable degree of AMR diversity was observed for both *S. dysenteriae* and *S. boydii*.

**Fig. 6:**
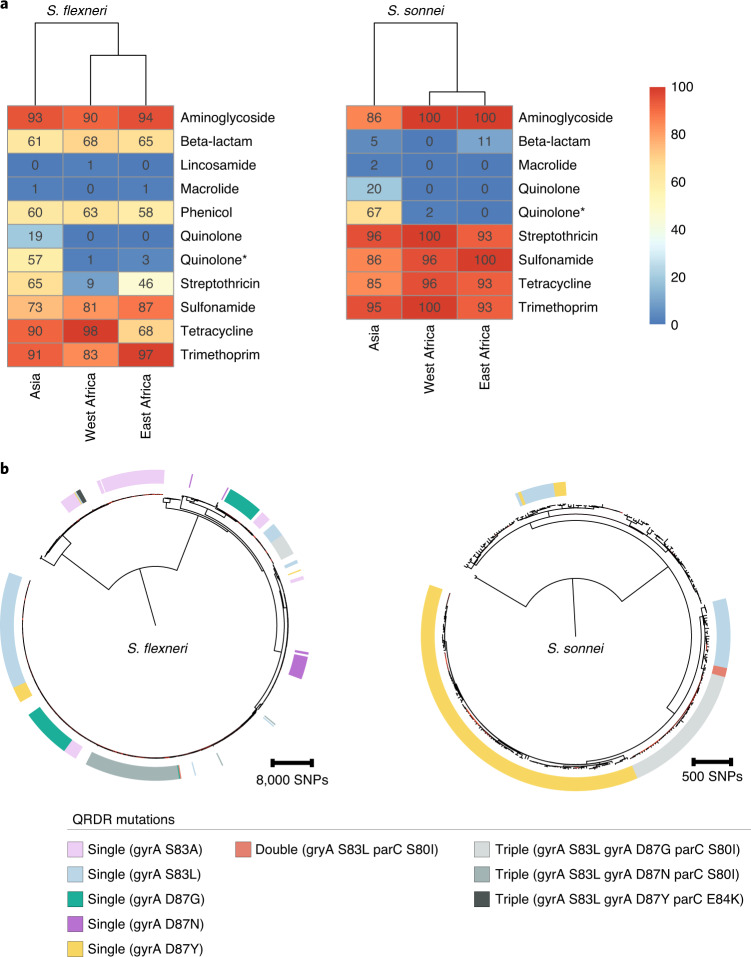
AMR genotypes grouped by region and convergent evolution of ciprofloxacin resistance. **a**, Detection of known AMR genetic determinants associated with drug class grouped by region. Each cell in the heatmap represents the percentage of isolates from a region containing genetic determinants associated with resistance to a drug class. Genetic determinant conferring reduced susceptibility to quinolone is indicated with an asterisk. **b**, The genetic convergent evolution of ciprofloxacin resistance in *S. flexneri* and *S. sonnei*. The presence of multiple monophyletic clades of QRDR mutations (single, double or triple according to the inlaid key) conferring reduced susceptibility or resistance to ciprofloxacin is shown in the outer ring. Figures for *S. boydii* and *S. dysenteriae* are shown in Supplementary Fig. 10. Source data

Overall, a high frequency of AMR genes conferring resistance against aminoglycoside, tetracycline, trimethoprim and sulfonamide antimicrobials was observed, while resistance against other antimicrobial classes varied with region and species (Fig. 6a and Supplementary Fig. 10a). The extended spectrum beta-lactamase gene *blaCTX-M-15* was detected in a small (9/1,246) percentage of isolates, and genes conferring resistance to macrolides and lincosamides were also infrequent (Extended Data Fig. 8), indicating that the recommended second-line treatments probably remain effective antimicrobials^47^.

However, higher rates of resistance were found against the first-line treatment. FQR in *Shigella* can be conferred through the acquisition of FQR genes or, more typically, by point mutations in the chromosomal Quinolone Resistance Determining Region (QRDR) within the DNA gyrase (*gryA*) and the topoisomerase IV (*parC*) genes. Single and double QRDR mutations are known to confer reduced susceptibility to ciprofloxacin and are evolutionary intermediates on the path to resistance, conferred by triple mutations in this region^45,48^. Overall, FQR genes were uncommon in *S. flexneri* (4%, 33/806), *S. sonnei* (1%, 3/305) and *S. dysenteriae* (7%, 4/60), but were present in 32% (24/75) of *S. boydii*. QRDR mutations were identified in all species (Extended Data Fig. 8), but were more common among *S. sonnei* (65%, 199/305) and *S. flexneri* (54%, 435/806) than among *S. boydii* (15%, 11/75) and *S. dysenteriae* (30%, 18/60). Among these, triple QRDR mutations were identified in 13% (106/806) of *S. flexneri* and 14% (44/305) of *S. sonnei*. Analysis of the QRDR mutants across the phylogenies indicates marked convergent evolution towards resistance across the genus. Specifically, all triple QRDR mutant *S. sonnei* belonged to one monophyletic subtype (previously described as globally emerging from Southeast Asia^49^), while three distinct triple QRDR mutational profiles were found across three polyphyletic *S. flexneri* genotypes (Fig. 6b). Thus, the polyphyletic distribution of single, double and triple QRDR mutants indicates continued convergent evolution of lineages with reduced susceptibility or increased resistance to FQR.

We then stratified the dataset by geographic region, which revealed that FQR was largely associated with isolates from Asia where fluoroquinolones are more frequently used compared with African sites (Fig. 6a and Supplementary Fig. 10a)^50^; this is consistent with trends observed in atypical enteropathogenic *Escherichia coli* isolated from GEMS^50^. Furthermore, analysis of African *Shigella* isolates from VIDA collected between 2015 and 2018 revealed that all species across West Africa and East Africa remained susceptible to ciprofloxacin^28^. Our analyses thus suggest that for the period of the GEMS trial (2007–2011), 17% (150/881) of *Shigella* isolates from Asia were resistant and 58% (508/881) had reduced susceptibility to the WHO-recommended antimicrobial. The high level of reduced susceptibility, together with marked convergent evolution towards resistance, suggests that management of shigellosis with fluroquinolones at these sites may soon be ineffective and regional antimicrobial treatment guidelines may require updating. These results indicate the value of AMR and genomic surveillance in LMICs for the control and management of shigellosis, and will be improved by initiatives such as the Africa Pathogen Genomics Initiative^51^ and the WHO Global Antimicrobial Resistance Surveillance System^52^.

## Conclusions

Pathogen genomics is a powerful tool that has a wide range of applications to help combat infectious diseases. Here we have applied this tool to an unparalleled systematically collected *Shigella* dataset to characterize the relevant population diversity of this pathogen across LMICs in a pre-vaccine era. This study has highlighted the urgent need to continue the development of *Shigella* vaccines for children in endemic areas. The genomic diversity in *Shigella* presents a major hurdle in controlling the disease and we have demonstrated the anticipated pitfalls of current vaccination approaches, emphasizing the importance of considering the local and global diversity of the pathogens in vaccine design and implementation. The relatively low heterogeneity among protein vaccine antigens in the *S. flexneri* population, and the lack of mutations predicted to be destabilizing, support the use of conserved antigens, and/or their inclusion alongside serotype-specific approaches for improved vaccine design. Our results also revealed that current antimicrobial treatment guidelines for shigellosis should be updated, particularly in Asia, and that improved and ongoing surveillance is essential to guide antimicrobial stewardship. Taken together, this study demonstrates the benefit of genomics in guiding prevention and control of shigellosis, providing further impetus to continue working to overcome the challenges associated with the implementation of WGS for pathogen surveillance in LMICs. Finally, our results suggest that annual *Shigella* surveillance would probably identify serotype switching, which would be especially important following the introduction of a vaccination programme. Although our results are focused on shigellosis, our approach is translatable to other bacterial pathogens and is particularly relevant as we enter the era of vaccines for AMR.

## Methods

### Dataset, bacterial isolates and sequencing

A total of 1,264 *Shigella* isolates from both cases and controls collected during GEMS were investigated in this study^2,3^. According to the GEMS study design, case enrolment required each child with diarrhoea (diarrhoea was defined as three or more loose stools within the previous 24 h) seeking care at a selected sentinel hospital or health centre to fulfill at least one of the criteria for MSD^2^. Controls were enrolled as children without diarrhoea, matched to every individual patient with MSD by age, sex and residential area. All isolates were derived from stool samples/rectal swabs: their identification, confirmation and isolation have been described previously^21^. A total of 1,344 isolates were sequenced at the Earlham Institute, with genomic DNA extraction, sequencing library construction and whole-genome sequencing carried out according to the Low Input Transposase Enabled (LITE) pipeline^53^. Among these, 225 isolates failed quality controls having mean sample depths of coverage <10× and an assembly sizes of <4 MB and were re-sequenced. For these isolates, genomic DNA was re-extracted at the University of Maryland School of Medicine (Baltimore, MD) from cultures grown in Lysogeny Broth overnight. DNA was extracted in 96-well format from 100 μl of sample using the MagAttract PowerMicrobiome DNA/RNA Kit (Qiagen) automated on a Hamilton Microlab STAR robotic platform. Bead disruption was conducted on a TissueLyser II (20 Hz for 20 min) instrument in a 96-well deep-well plate in the presence of 200 μl phenol/chloroform. Genomic DNA was eluted in 90 μl water after magnetic bead clean up and the resulting genomic DNA was quantified by Pico Green. The genomic DNA was shipped to the Centre for Genomic Research (CGR, University of Liverpool) for whole-genome sequencing. Sequencing library was constructed using NEBNext Ultra II FS DNA Library Prep Kit for Illumina and sequenced on the Illumina NovaSeq 6000 platform, generating 150 bp paired-end reads.

An additional 125 publicly available *Shigella* and *E. coli* reference genomes were included in the phylogenetic analyses and a further 236 *S. flexneri* genomes were included in the assessment of vaccine protein antigens. Details of GEMS and reference genomes analysed in this study are listed in Supplementary Tables 2 and 3, respectively.

### Sequence mapping and variant calling

Adaptors and low-quality bases were trimmed with Trimmomatic v0.38^54^, and reads qualities were assessed using FastQC v0.11.6 (https://www.bioinformatics.babraham.ac.uk/projects/fastqc/) and MultiQC v1.7^55^. Filtered reads were mapped against *Shigella* reference genomes with BWA mem v0.7.17^56^ using default parameters. *S. flexneri*, *S. sonnei*, *S. boydii* and *S. dysenteriae* sequencing reads were mapped against reference genomes from Sf2a strain 301 (accession NC_004337), Ss046 (accession NC_007384), Sb strain CDC 3083-94 (accession NC_010658) and Sd197 (accession NC_007606), respectively. Mappings were filtered and sorted using the SAMtools suite v1.9-47^57^, and optical duplicate reads were marked using Picard v2.21.1-SNAPSHOT MarkDuplicates (http://broadinstitute.github.io/picard/). QualiMap v2.2.2^58^ was used to evaluate mapping qualities and estimate mean sample depth of coverage. Sequencing reads for isolates sequenced using the LITE pipeline and re-sequenced at the CGR were combined to increase overall sample depth of coverage. Sequence variants were identified against reference using SAMtools v1.9-47 mpileup and bcftools v1.9-80^57^. Low-quality SNPs were filtered if mapping quality was <60, Phred-scaled quality score was <30 and read depth was <4.

### Phylogenetic reconstruction, inference of genomic diversity, and genotyping

Filtered SNP variants were used to generate a reference-based pseudogenome for each sample, where regions with depth of coverage >4× were masked in the pseudogenome. Additionally, regions containing phage (identified using PHASTER^59^ web server) and insertion sequences were identified from the reference genomes, and co-ordinates were used to mask these sites on the pseudogenomes using BEDTools v2.28.0 maskfasta^60^. For each species, chromosome sequences from the masked pseudogenomes were extracted and concatenated. Gubbins v2.3.4^61^ was used to remove regions of recombination and invariant sites from the concatenated pseudogenomes (Supplementary Fig. 4). This generated a chromosomal SNP alignment length of 78,251 bp for *S. flexneri* (*n* = 806), 5,081 bp for *S. sonnei* (*n* = 305), 98,842 bp for *S. boydii* (*n* = 75) and 45,031 bp for *S. dysenteriae* (*n* = 60). Maximum-likelihood phylogenetic reconstruction was performed independently for each species and inferred with IQ-TREE v2.0-rc2^62^ using the FreeRate nucleotide substitution, invariable site and ascertainment bias correction model, with 1,000 bootstrap replicates. To contextualize GEMS isolates within the established genomic subtypes and to infer the most appropriate root for each species tree, phylogenetic trees were reconstructed including publicly available reference genomes of isolates from previously defined lineages/phylogroups/clades and *E. coli* isolates (Supplementary Table 3). The phylogenetic trees for *S. flexneri*, *S. boydii* and *S. dysenteriae* waere rooted using *E. coli* strain IAI1-117 (accession SRR2169557) as an outgroup. The phylogenetic tree for *S. sonnei* was midpoint rooted. Visualizations were performed using interactive Tree of Life (iTOL) v6.1.1^63^. Assignment of *S. sonnei* genomes to hierarchical genotypes was performed using the script sonnei_genotype.py (https://github.com/katholt/sonneityping) on the basis of mapping files, and according to a previously described genotyping scheme^26^.

To measure the extent of *Shigella* genomic diversity among the GEMS population, pairwise SNP distance was determined from the alignment of core genome SNPs identified outside regions of recombination using snp-dists v0.7.0 (https://github.com/tseemann/snp-dists). For each species, the genomic diversity, measured by SNPs per kbp, was determined by dividing the core genome SNP alignment length by the core genome size (*S. flexneri* 4,015,307 bp, *S. sonnei* 4,177,070 bp, *S. boydii* 4,088,693 bp and *S. dysenteriae* 3,821,602 bp). To scale the proportion of disease burden attributable to the genome diversity of each species, the percentage of species contribution to GEMS shigellosis disease burden was divided by the number of SNPs per kbp.

### Serotype switching timeframe inference

**Extended Data Fig. 10:**
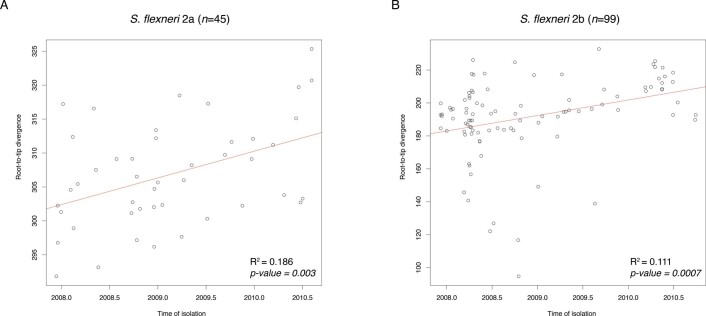
Temporal phylogenetic signal for *S. flexneri*. Correlation between isolate sampling time in months (x-axis) and phylogenetic root-to-tip divergence (y-axis), as estimated by TempEst based on ML phylogeny of each subclade. The two datasets correspond to *S. flexneri* serotype 2a isolates belonging to node A (A) and *S. flexneri* serotype 2b isolates belonging to node B (B) from PG3 in Supplementary Fig. 12. The linear regression line is coloured in red, with the coefficient of determination (R^2^) and *p*-value displayed for each plot. Source data

To estimate the probable timeframe of serotype switching, we performed temporal phylogenetic reconstruction to infer the time of divergence along branches exhibiting serotype switching. We streamlined the analysis and focused on isolates belonging to two subclades of *S. flexneri* PG3. First, for each of the two subclades (*n* = 99 and *n* = 45), a maximum-likelihood phylogeny was reconstructed on the basis of genome multiple sequence alignments (described above). Then, TempEst v1.5.3^64^ was used to determine whether there was sufficient temporal signal in the data by inferring linear relationship between root-to-tip distances of the phylogenetic branches and the year of sample isolation. Data from both subclades revealed positive correlation between sampling time and phylogenetic root-to-tip divergence, with *R*^2^ of 0.186 and 0.111 (Extended Data Fig. 10). Once temporal signals within each of the two datasets were confirmed, core genome SNP alignments of length 559 bp and 1,244 bp were analysed independently using BEAST2 v2.6.1^65^. The parameters were as follows: dates specified as days, bModelTest^66^ implemented in BEAST2 was used to infer the most appropriate substitution model, a relaxed log normal clock rate with a coalescent Bayesian skyline model for population growth. Beauti v2.6.3^65^ was used to general xml configuration files. A total of five independent chains were performed, each with chain length of 250,000,000, logging every 1,000 and accounting for invariant sites. Convergence of each run was visually assessed with Tracer v1.7.1^67^, with all parameter effective sampling sizes ≥200. Tree files were sampled and combined using LogCombiner v2.6.1, the combined files were then summarized using TreeAnnotator v2.6.0 with 10% burn-in to generate the Maximum Clade Credibility tree^68^. Divergence time was inferred by reading the branch length from the most recent common ancestor to the first sampled isolate that serotype-switched.

### Genome assembly and annotation

Draft genome sequences were assembled using Unicycler v0.4.7^69^ with –min_fasta_length set to 200. QUAST v5.0.2^70^ was used to assess the qualities of the assemblies. Assemblies with total assembly length outside the range of <4 Mbp and >6.4 Mbp were removed, resulting in an average length of 4,275,508 bp (range 4,004,109–4,538,734 bp) for *S. flexneri*, 4,264,097 bp (range 4,008,630–4,779,279 bp) for *S. sonnei*, 4,227,671 bp (range 4,000,714–4,689,815 bp) for *S. boydii* and 4,297,921 bp (range 4,040,642–4,659,860 bp) for *S. dysenteriae*. An average N50 value of 29,804 bp (range 6,810–34,658 bp) was generated for *S. flexneri*, 23,961 bp (range 11,547–30,008 bp) for *S. sonnei*, 20,835 bp (range 15,323–40,119 bp) for *S. boydii* and 22,137 bp (range 14,090–31,358 bp) for *S. dysenteriae*. Draft genomes were annotated using Prokka v1.13.3^71^.

### Pangenome analysis

The pangenome of each species was defined using Roary v3.12.0^72^ without splitting paralogues. The pangenome accumulation curves were generated separately for each species using the specaccum function from Vegan v2.5-7 (https://github.com/vegandevs/vegan/), with 100 permutations and random subsampling. Inspections of the variable gene content showed that all four species had open pangenomes, implying that the number of unique genes increases with the addition of newly sequenced genomes.

### *Shigella flexneri* molecular serotyping

*Shigella* serotype data were provided by collaborators at the University of Maryland School of Medicine; serotyping was performed as previously described^21^. In silico serotyping of *S. flexneri* genomes was performed using ShigaTyper v1.0.6^73^, which detects the presence of serotype-determining genetic elements from sequencing reads to predict serotype. ShigaTyper predictions were 84% concordant with the serotype data provided. SRST2 v2^74^ was used to detect mutations within serotype-determining genetic elements, and was run against ShigaTyper sequence database with default parameters.

### Protein antigen screening

To determine the presence of antigen vaccine candidates among GEMS *Shigella* isolates, genes of the antigen vaccine candidates were screened against draft genome assemblies using screen_assembly^19^, with a threshold of ≥80% identity and ≥70% coverage to the reference sequence. Reference sequences for *ipaB*, *ipaC*, *ipaD* and *icsP* were derived from *S. flexneri* 5a strain M90T (accession GCA_004799585) and those for *ompA* and *sigA* were derived from *S. flexneri* 2a strain 2457 T (accession NC_004741), both strains being commonly used in the laboratory for vaccine development. Antigen sequence variations were determined by examining the BLASTp^75^ percentage identity against relevant query reference sequences. Allelic variations of antigen vaccine candidates among the *S. flexneri* population were identified manually by visualizing amino acid sequence alignments using AliView v1.26^76^. Publicly available *S. flexneri* genomes were also integrated into the analysis, with assembled genomes downloaded from EnteroBase (accessed 25 August 2021), including all isolates sampled between 2007 and 2021 from across LMICs (Asia *n* = 155 and Africa *n* = 81). No samples from Latin America met these criteria.

### Protein antigen modelling

To assess the effect of point mutations on protein stability and vaccine escape, six antigen candidates from *S. flexneri* PG3 were modelled: OmpA, SigA, IcsP, IpaB, IpaC and IpaD (Supplementary Table 1). PG3 was selected as it is the most prevalent phylogroup and is therefore the target of current vaccine development. To model the antigen targets, we first searched for a suitable template using HHPred^77,78^. Five of the six proteins (OmpA, SigA, IcsP, IpaB and IpaD) had suitable homologues available. To improve the performance of the comparative modelling, the signal peptides for OmpA, SigA and IcsP were removed and OmpA, SigA and IpaB were modelled in two parts to make use of optimal templates. RosettaCM source release-188^79^ was used to generate 200 models for each of the five proteins using the single best available template. For IpaC, where no suitable templates were available, trRosetta^80^ was used to create five de novo predicted models. The best model for each antigen candidate was selected using QMEAN’s v4.2.0 average local score. QMEANbrane v4.2.0^81,82^ was used for suitable membrane proteins (IpaB, IpaC and IpaD), otherwise QMEANDisCo v4.2.0^81^ was used (Supplementary Table 7). Full details of the modelling and ranking are shown in Supplementary Table 8. The effect of point mutations on the stability of the antigen candidates was assessed using PremPS^83^, and the default criterion of ΔΔG > 1 kcal mol^−1^ was used to define highly destabilizing mutations.

### Detection of AMR genetic determinants and AMR testing

To detect the presence of known genetic determinants for AMR, AMRFinderPlus v3.9.3^84^ was used to screen draft genome assemblies against the AMRFinderPlus database, which is derived from the Pathogen Detection Reference Gene Catalog (https://www.ncbi.nlm.nih.gov/pathogens/). AMRFinderPlus was performed with the organism-specific option for *Escherichia*, to screen for both point mutations and genes, and filter out uninformative genes that were nearly universal in a group. The output was then filtered to remove genetic determinants identified with ≤80% coverage and ≤90% identity. The presence of *S. sonnei* virulence plasmid was confirmed using short-read mapping using BWA mem (as described above) against the reference virulence plasmid from Ss046 (GenBank accession CP000039.1). Presence of the plasmid was defined by the mapping of >60% breadth of coverage across the reference. Visualizations of AMR resistance profiles were performed with UpSetR v2.1.3^85^. Four *S. flexneri* isolates with triple QRDR mutations were phenotypically tested for ciprofloxacin resistance using the Kirby–Bauer standardized disk diffusion method^86^.

### Statistical analyses

The strength of association of *S. flexneri* genomic subtype and serotype with the occurrence of case outcome was calculated using MedCalc Software odds ratio calculator v20 (https://www.medcalc.org/calc/odds_ratio.php) to report the odds ratio, 95% confidence interval and statistical association. Association of genomic subtype with serotype and serotype switching was tested using Fisher’s exact test. Linear regression analysis was used to determine the correlation between serotype diversity and various properties of genomic subtype. Both analyses were performed using R v4.0.3.

### Reporting Summary

Further information on research design is available in the Nature Research Reporting Summary linked to this article.

### Reporting Summary

Further information on research design is available in the Nature Research Reporting Summary linked to this article.

## Supplementary information

Supplementary Information Supplementary Figs. 1–10 and Tables 1, 4, 6 and 7.

Reporting Summary

Peer Review Information

Supplementary Table Additional supplementary files.

Supplementary Data Input file consisting of pairwise SNP distances among different serotypes for the generation of Supplementary Fig. 1b.

Supplementary Data Input file consisting of pairwise SNP distances among different *S. flexneri* PGs and *S. sonnei* isolates for the generation of Supplementary Fig. 2.

Supplementary Data Input file consisting of BLASTp results for the generation of Supplementary Fig. 7.

Supplementary Data Input file consisting of amino acid variation locations and frequencies among the six protein vaccine candidates for the generation of Supplementary Fig. 8.

## Data Availability

Short-read sequences supporting the findings of this study have been deposited in the European Nucleotide Archive (https://www.ebi.ac.uk/ena/) under the project accession number PRJEB45383. Accession numbers for isolates used in this study are listed in Supplementary Table 2. Publicly available sequences were downloaded from GenBank (https://www.ncbi.nlm.nih.gov/genbank/), Sequence Read Archive (https://www.ncbi.nlm.nih.gov/sra), the European Nucleotide Archive (https://www.ebi.ac.uk/ena) and EnteroBase (http://enterobase.warwick.ac.uk/). Accession numbers of publicly available genomes are listed in Supplementary Table 3. Phylogenetic trees, antigen protein models and BEAST input and output files have been deposited in FigShare (10.6084/m9.figshare.14743833). Source data are provided with this paper.

## Source data

Source Data Fig. 1 Input file consisting of pairwise SNP distances for the generation of Fig. 1.
Source Data Fig. 5 Input file consisting of AMR profiles observed among *Shigella* for the generation of Fig. 5.
Source Data Fig. 6 Input files consisting of AMR classes observed among *Shigella* across various regions for the generation of Fig. 6 and Supplementary Fig. 10.
Source Data Extended Data Fig. 1 Input binary files consisting of genes presence and absence for the generation of Extended Data 1.
Source Data Extended Data Fig. 2 Gubbins output gff files for the generation of Extended Data Fig. 2.
Source Data Extended Data Fig. 3 Input file consisting of pairwise SNP distances among *Shigella* across difference GEMS regions for the generation of Extended Data 3.
Source Data Extended Data Fig. 4 Input file listing the numbers of serotypes across different GEMS regions for the generation of Extended Data 4.
Source Data Extended Data Fig. 5 Input files comparing serotype diversity and genetic diversity between the six *S. flexneri* PGs for the generation of Extended Data 5.
Source Data Extended Data Fig. 8 Input files consisting of the numbers and frequencies of AMR genes detected in each *Shigella* species for the generation of Extended Data 8.
Source Data Extended Data Fig. 9 Input binary files consisting of predicted resistance/reduced susceptibility against various AMR drug classes among *Shigella* species for the generation of Extended Data 9.
Source Data Extended Data Fig. 10 Input files consisting of TempEst output for the generation of Extended Data 10.

## Code availability

All codes used in this study are described in the Methods. No custom algorithms were used for analyses.

## References

[1] Khalil IA (2018). Morbidity and mortality due to *Shigella* and enterotoxigenic *Escherichia coli* diarrhoea: the Global Burden of Disease Study 1990–2016. Lancet Infect. Dis..

[2] Kotloff KL (2013). Burden and aetiology of diarrhoeal disease in infants and young children in developing countries (the Global Enteric Multicenter Study, GEMS): a prospective, case-control study. Lancet.

[3] Liu J (2016). Use of quantitative molecular diagnostic methods to identify causes of diarrhoea in children: a reanalysis of the GEMS case-control study. Lancet.

[4] Kotloff KL, Riddle MS, Platts-Mills JA, Pavlinac P, Zaidi AKM (2018). Shigellosis. Lancet.

[5] Shrivastava SR, Shrivastava PS, Ramasamy J (2018). World health organization releases global priority list of antibiotic-resistant bacteria to guide research, discovery, and development of new antibiotics. J. Med. Soc..

[6] Barry EM (2013). Progress and pitfalls in *Shigella* vaccine research. Nat. Rev. Gastroenterol. Hepatol..

[7] Cohen D, Green MS, Block C, Slepon R, Ofek I (1991). Prospective study of the association between serum antibodies to lipopolysaccharide O antigen and the attack rate of shigellosis. J. Clin. Microbiol..

[8] Ferreccio C (1991). Epidemiologic patterns of acute diarrhea and endemic *Shigella* infections in children in a poor periurban setting in Santiago, Chile. Am. J. Epidemiol..

[9] Formal SB (1991). Effect of prior infection with virulent *Shigella flexneri* 2a on the resistance of monkeys to subsequent infection with *Shigella sonnei*. J. Infect. Dis..

[10] Kotloff KL (1995). A modified *Shigella* volunteer challenge model in which the inoculum is administered with bicarbonate buffer: clinical experience and implications for *Shigella* infectivity. Vaccine.

[11] Levine MM, Kotloff KL, Barry EM, Pasetti MF, Sztein MB (2007). Clinical trials of *Shigella* vaccines: two steps forward and one step back on a long, hard road. Nat. Rev. Microbiol..

[12] Mani S, Wierzba T, Walker RI (2016). Status of vaccine research and development for *Shigella*. Vaccine.

[13] Talaat KR (2021). Human challenge study with a *Shigella* bioconjugate vaccine: analyses of clinical efficacy and correlate of protection. EBioMedicine.

[14] Passwell JH (2010). Age-related efficacy of *Shigella* O-specific polysaccharide conjugates in 1–4-year-old Israeli children. Vaccine.

[15] Turbyfill KR, Kaminski RW, Oaks EV (2008). Immunogenicity and efficacy of highly purified invasin complex vaccine from *Shigella flexneri* 2a. Vaccine.

[16] Martinez-Becerra FJ (2012). Broadly protective *Shigella* vaccine based on type III secretion apparatus proteins. Infect. Immun..

[17] Berlanda Scorza F (2012). High yield production process for *Shigella* outer membrane particles. PLoS ONE.

[18] Frenck RW (2021). Efficacy, safety, and immunogenicity of the *Shigella sonnei* 1790GAHB GMMA candidate vaccine: results from a phase 2b randomized, placebo-controlled challenge study in adults. EClinicalMedicine.

[19] Davies MR (2019). Atlas of group A streptococcal vaccine candidates compiled using large-scale comparative genomics. Nat. Genet..

[20] Telford JL (2008). Bacterial genome variability and its impact on vaccine design. Cell Host Microbe.

[21] Livio S (2014). *Shigella* isolates from the global enteric multicenter study inform vaccine development. Clin. Infect. Dis..

[22] Connor TR (2015). Species-wide whole genome sequencing reveals historical global spread and recent local persistence in *Shigella flexneri*. Elife.

[23] Holt KE (2012). *Shigella sonnei* genome sequencing and phylogenetic analysis indicate recent global dissemination from Europe. Nat. Genet..

[24] Njamkepo E (2016). Global phylogeography and evolutionary history of *Shigella dysenteriae* type 1. Nat. Microbiol..

[25] Kania DA, Hazen TH, Hossain A, Nataro JP, Rasko DA (2016). Genome diversity of *Shigella boydii*. Pathog. Dis..

[26] Hawkey J (2021). Global population structure and genotyping framework for genomic surveillance of the major dysentery pathogen, *Shigella sonnei*. Nat. Commun..

[27] Sahl JW (2015). Defining the phylogenomics of *Shigella* species: a pathway to diagnostics. J. Clin. Microbiol..

[28] Badji, H. et al. Prevalence, antimicrobial resistance, and distribution of *Shigella* among children under five in three sub-Saharan African countries in the Vaccine Impact on Diarrhea in Africa Study. in *American Society of Tropical Medicine and Hygiene*.

[29] von Seidlein L (2006). A multicentre study of *Shigella* diarrhoea in six Asian countries: disease burden, clinical manifestations, and microbiology. PLoS Med..

[30] Ye C (2010). Emergence of a new multidrug-resistant serotype X variant in an epidemic clone of *Shigella flexneri*. J. Clin. Microbiol..

[31] Allison GE, Verma NK (2000). Serotype-converting bacteriophages and O-antigen modification in *Shigella flexneri*. Trends Microbiol..

[32] Sun Q (2012). A novel plasmid-encoded serotype conversion mechanism through addition of phosphoethanolamine to the O-antigen of *Shigella flexneri*. PLoS ONE.

[33] Weinberger DM, Malley R, Lipsitch M (2011). Serotype replacement in disease after pneumococcal vaccination. Lancet.

[34] Brueggemann AB, Pai R, Crook DW, Beall B (2007). Vaccine escape recombinants emerge after pneumococcal vaccination in the United States. PLoS Pathog..

[35] Riddle MS (2011). Safety and immunogenicity of an intranasal *Shigella flexneri* 2a Invaplex 50 vaccine. Vaccine.

[36] McVicker G, Tang CM (2016). Deletion of toxin-antitoxin systems in the evolution of *Shigella sonnei* as a host-adapted pathogen. Nat. Microbiol.

[37] Garcia-Beltran, W. F. et al. Multiple SARS-CoV-2 variants escape neutralization by vaccine-induced humoral immunity. *Cell* **184**, 2523 (2021). 10.1016/j.cell.2021.04.006 PMC8082941 33930298

[38] Zhou, D. et al. Evidence of escape of SARS-CoV-2 variant B.1.351 from natural and vaccine-induced sera. *Cell* **184**, 2348-2361 (2021). 10.1016/j.cell.2021.02.037 PMC7901269 33730597

[39] Mills JA, Buysse JM, Oaks EV (1988). *Shigella flexneri* invasion plasmid antigens B and C: epitope location and characterization with monoclonal antibodies. Infect. Immun..

[40] Turbyfill KR, Mertz JA, Mallett CP, Oaks EV (1998). Identification of epitope and surface-exposed domains of *Shigella flexneri* invasion plasmid antigen D (IpaD). Infect. Immun..

[41] Czerkinsky, C. & Kim, D. W. *Shigella* protein antigens and methods. US patent 8168203 (2012).

[42] Pore D, Mahata N, Pal A, Chakrabarti MK (2011). Outer membrane protein A (OmpA) of *Shigella flexneri* 2a induces protective immune response in a mouse model. PLoS ONE.

[43] *Guidelines for the Control of Shigellosis, Including Epidemics Due to Shigella dysenteriae type 1* (WHO, 2005).

[44] Chung The H, Baker S (2018). Out of Asia: the independent rise and global spread of fluoroquinolone-resistant *Shigella*. Microb. Genom..

[45] Sadouki Z (2017). Comparison of phenotypic and WGS-derived antimicrobial resistance profiles of *Shigella sonnei* isolated from cases of diarrhoeal disease in England and Wales, 2015. J. Antimicrob. Chemother..

[46] Baker KS (2015). Intercontinental dissemination of azithromycin-resistant shigellosis through sexual transmission: a cross-sectional study. Lancet Infect. Dis..

[47] Williams PCM, Berkley JA (2018). Guidelines for the treatment of dysentery (shigellosis): a systematic review of the evidence. Paediatr. Int. Child Health.

[48] Chung The H (2016). South Asia as a reservoir for the global spread of Ciprofloxacin-resistant *Shigella sonnei*: a cross-sectional study. PLoS Med..

[49] Chung The H (2019). Dissecting the molecular evolution of fluoroquinolone-resistant *Shigella sonnei*. Nat. Commun..

[50] Ingle DJ, Levine MM, Kotloff KL, Holt KE, Robins-Browne RM (2018). Dynamics of antimicrobial resistance in intestinal *Escherichia coli* from children in community settings in South Asia and sub-Saharan Africa. Nat. Microbiol.

[51] Makoni M (2020). Africa’s $100-million pathogen genomics initiative. Lancet Microbe.

[52] N.G.H.R.U.O.G.S.O. AMR (2020). Whole-genome sequencing as part of national and international surveillance programmes for antimicrobial resistance: a roadmap. BMJ Glob. Health.

[53] Perez-Sepulveda, B. M. et al. An accessible, efficient and global approach for the large-scale sequencing of bacterial genomes. *Genome Biol*. **22**, 349 (2021). 10.1186/s13059-021-02536-3 PMC8690886 34930397

[54] Bolger AM, Lohse M, Usadel B (2014). Trimmomatic: a flexible trimmer for Illumina sequence data. Bioinformatics.

[55] Ewels P, Magnusson M, Lundin S, Kaller M (2016). MultiQC: summarize analysis results for multiple tools and samples in a single report. Bioinformatics.

[56] Li, H. Aligning sequence reads, clone sequences and assembly contigs with BWA-MEM. Preprint at https://arxiv.org/abs/1303.3997 (2013).

[57] Li H (2009). The sequence alignment/map format and SAMtools. Bioinformatics.

[58] Garcia-Alcalde F (2012). Qualimap: evaluating next-generation sequencing alignment data. Bioinformatics.

[59] Arndt D (2016). PHASTER: a better, faster version of the PHAST phage search tool. Nucleic Acids Res..

[60] Quinlan AR (2014). BEDTools: the Swiss-Army tool for genome feature analysis. Curr. Protoc. Bioinformatics.

[61] Croucher NJ (2015). Rapid phylogenetic analysis of large samples of recombinant bacterial whole genome sequences using Gubbins. Nucleic Acids Res..

[62] Nguyen LT, Schmidt HA, von Haeseler A, Minh BQ (2015). IQ-TREE: a fast and effective stochastic algorithm for estimating maximum-likelihood phylogenies. Mol. Biol. Evol..

[63] Letunic I, Bork P (2019). Interactive Tree Of Life (iTOL) v4: recent updates and new developments. Nucleic Acids Res..

[64] Rambaut A, Lam TT, Max Carvalho L, Pybus OG (2016). Exploring the temporal structure of heterochronous sequences using TempEst (formerly Path-O-Gen). Virus Evol..

[65] Bouckaert R (2014). BEAST 2: a software platform for Bayesian evolutionary analysis. PLoS Comput. Biol..

[66] Bouckaert RR, Drummond AJ (2017). bModelTest: Bayesian phylogenetic site model averaging and model comparison. BMC Evol. Biol..

[67] Rambaut A, Drummond AJ, Xie D, Baele G, Suchard MA (2018). Posterior summarization in Bayesian phylogenetics using Tracer 1.7. Syst. Biol..

[68] Bouckaert R (2019). BEAST 2.5: an advanced software platform for Bayesian evolutionary analysis. PLoS Comput. Biol..

[69] Wick RR, Judd LM, Gorrie CL, Holt KE (2017). Unicycler: resolving bacterial genome assemblies from short and long sequencing reads. PLoS Comput. Biol..

[70] Gurevich A, Saveliev V, Vyahhi N, Tesler G (2013). QUAST: quality assessment tool for genome assemblies. Bioinformatics.

[71] Seemann T (2014). Prokka: rapid prokaryotic genome annotation. Bioinformatics.

[72] Page AJ (2015). Roary: rapid large-scale prokaryote pan genome analysis. Bioinformatics.

[73] Wu, Y., Lau, H. K., Lee, T., Lau, D. K. & Payne, J. In silico serotyping based on whole-genome sequencing improves the accuracy of *Shigella* identification. *Appl. Environ. Microbiol*. **85** e00165-19 (2019). 10.1128/AEM.00165-19 PMC6585509 30709819

[74] Inouye M (2014). SRST2: rapid genomic surveillance for public health and hospital microbiology labs. Genome Med..

[75] Altschul SF (1997). Gapped BLAST and PSI-BLAST: a new generation of protein database search programs. Nucleic Acids Res..

[76] Larsson A (2014). AliView: a fast and lightweight alignment viewer and editor for large datasets. Bioinformatics.

[77] Hildebrand A, Remmert M, Biegert A, Soding J (2009). Fast and accurate automatic structure prediction with HHpred. Proteins.

[78] Zimmermann L (2018). A completely reimplemented MPI bioinformatics toolkit with a new HHpred server at its core. J. Mol. Biol..

[79] Song Y (2013). High-resolution comparative modeling with RosettaCM. Structure.

[80] Yang J (2020). Improved protein structure prediction using predicted interresidue orientations. Proc. Natl Acad. Sci. USA.

[81] Studer G (2020). QMEANDisCo-distance constraints applied on model quality estimation. Bioinformatics.

[82] Studer G, Biasini M, Schwede T (2014). Assessing the local structural quality of transmembrane protein models using statistical potentials (QMEANBrane). Bioinformatics.

[83] Chen Y (2020). PremPS: predicting the impact of missense mutations on protein stability. PLoS Comput. Biol..

[84] Feldgarden, M. et al. Validating the AMRFinder tool and resistance gene database by using antimicrobial resistance genotype–phenotype correlations in a collection of isolates. *Antimicrob. Agents Chemother*. **63**, e00483-19 (2019). 10.1128/AAC.00483-19 PMC6811410 31427293

[85] Conway JR, Lex A, Gehlenborg N (2017). UpSetR: an R package for the visualization of intersecting sets and their properties. Bioinformatics.

[86] Hudzicki, J. *Kirby–Bauer Disk Diffusion Susceptibility Test Protocol* (American Society for Microbiol, 2009).

