# Supplementary material for: Pathogenomic analyses of Shigella isolates inform factors limiting shigellosis prevention and control across LMICs
Source: Nat Microbiol. 2022 Jan 31;7(2):251–61. doi: 10.1038/s41564-021-01054-z (PMC8813619; doi:10.1038/s41564-021-01054-z)
Supplement: Supplementary file 1 — Supplementary Figs. 1–10 and Tables 1, 4, 6 and 7. [file 41564_2021_1054_MOESM1_ESM.pdf]

nature portfolio

https://doi.org/10.1038/s41564-021-01054-z

Supplementary information

Pathogenomic analyses of *Shigella* isolates inform factors limiting shigellosis prevention and control across LMICs

In the format provided by the authors and unedited

A

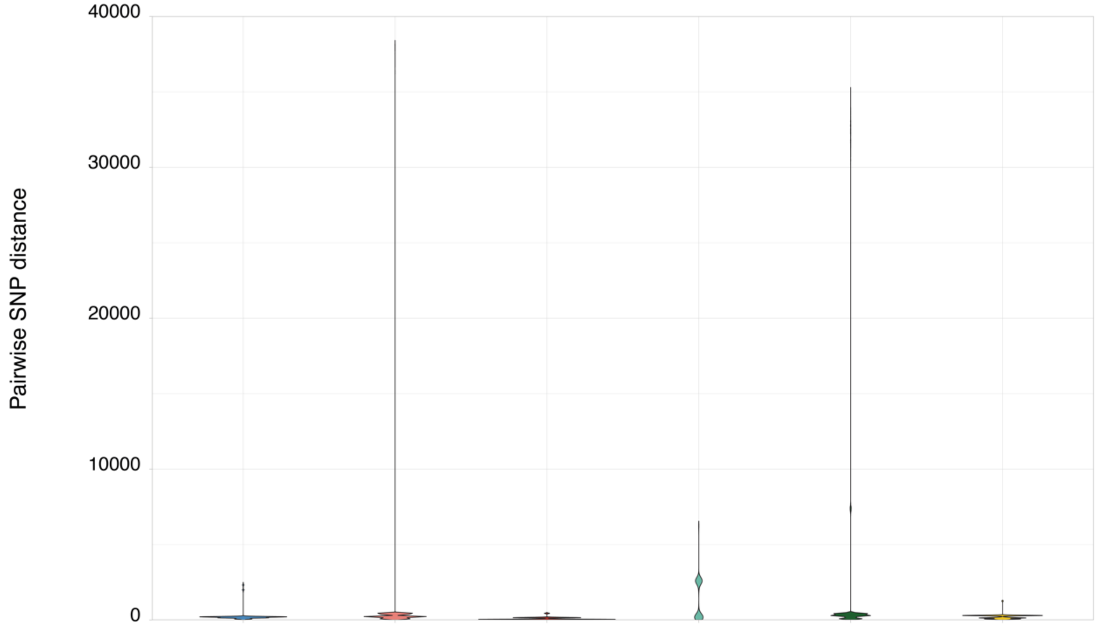

Serotype/subserotype	*S. flexneri 1b*	*S. flexneri 2a*	*S. flexneri 2b*	*S. flexneri 3a*	*S. flexneri 6*	*S. sonnei*
Genomic diversity (SNPs / kbp)	1.3	11.2	0.6	3.1	12.3	1.2 (0.9)
Attributable shigellosis (%)	7.7	20.4	11.0	9.3	11.1	23.9
Attributable shigellosis / Genomic diversity	5.9	1.8	18.4	3.0	0.9	19.9 (26.6)

B

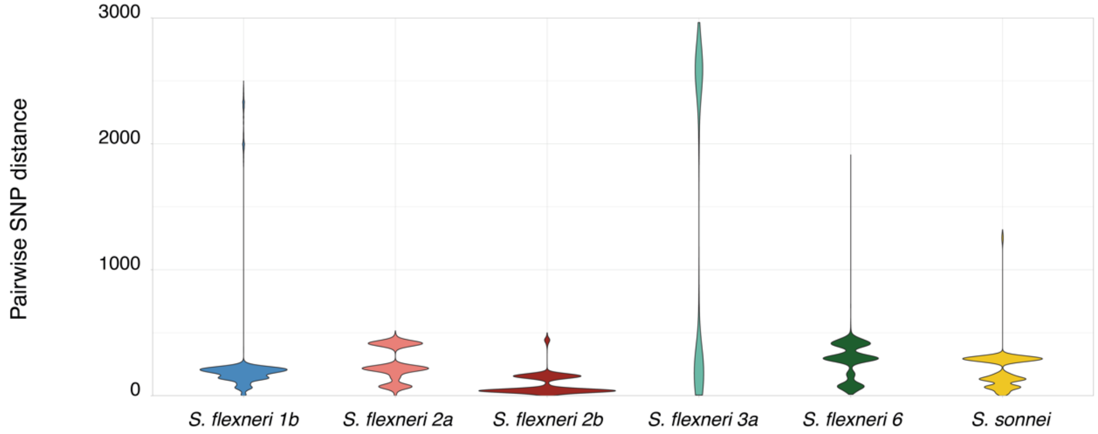

Supplementary Figure. 1

Genomic diversity among the six most common *Shigella* serotypes/subserotypes in GEMS and responsible for attributing the highest shigellosis. (A) Pairwise SNP distances (y-axis) among *Shigella* isolates within serotype/subserotype. Table below the plots demonstrates for each serotypes/subserotypes the genomic diversity, the contribution to GEMS shigellosis burden and the shigellosis burden relative to genomic diversity. (B) Higher resolution violin plots displaying diveristy among *Shigella* isolates within serotype/subserotype, with outliers excluded at a threshold of 3000 SNPs.

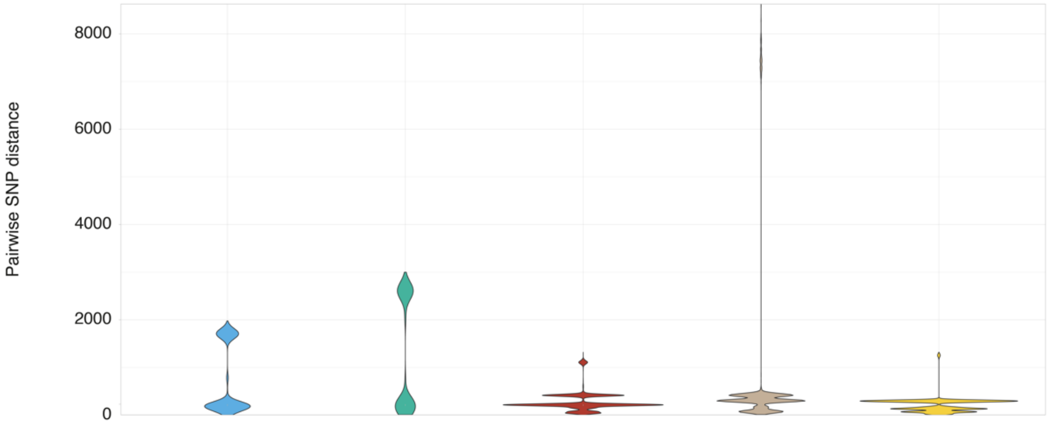

Genomic subtype	PG1	PG2	PG3	Sf6	*S. sonnei*
Genomic diversity (SNPs / kbp)	1.7	1.8	2.6	3.5	1.2 (0.9)
Attributable shigellosis (%)	14.4	12.0	42.7	14.4	23.9
Attributable shigellosis / Genomic diversity	8.5	6.7	16.4	4.1	19.9 (26.6)

Supplementary Figure. 2

Genomic diversity among the five most common *Shigella* genomic subtypes in GEMS.

A

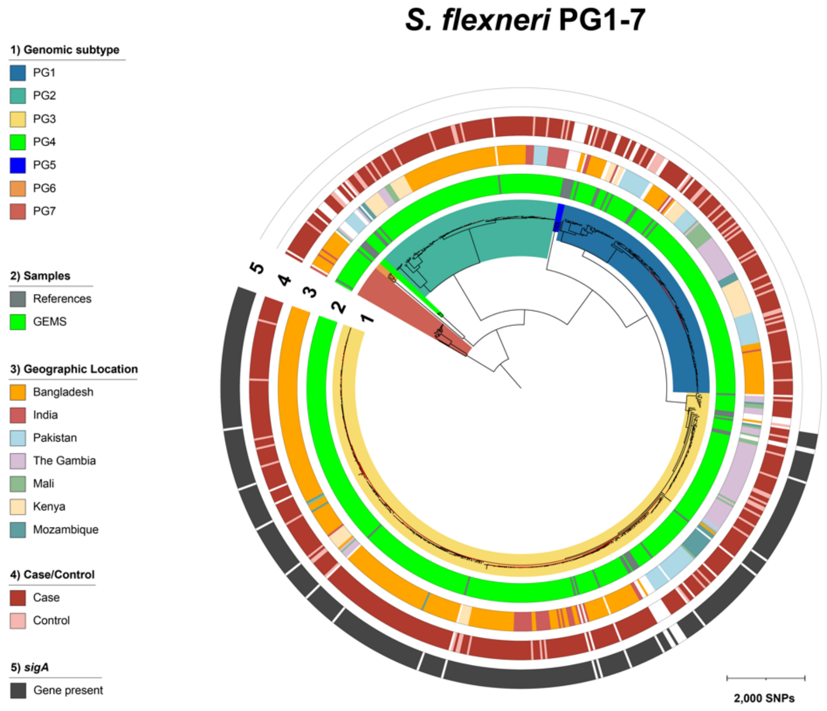

S. flexneri PG1-7
1) Genomic subtype
PG1
PG2
PG3
PG4
PG5
PG6
PG7
2) Samples
References
GEMS
3) Geographic Location
Bangladesh
India
Pakistan
The Gambia
Mali
Kenya
Mozambique
4) Case/Control
Case
Control
5) sigA
Gene present
1
2
3
4
5
2,000 SNPs

B

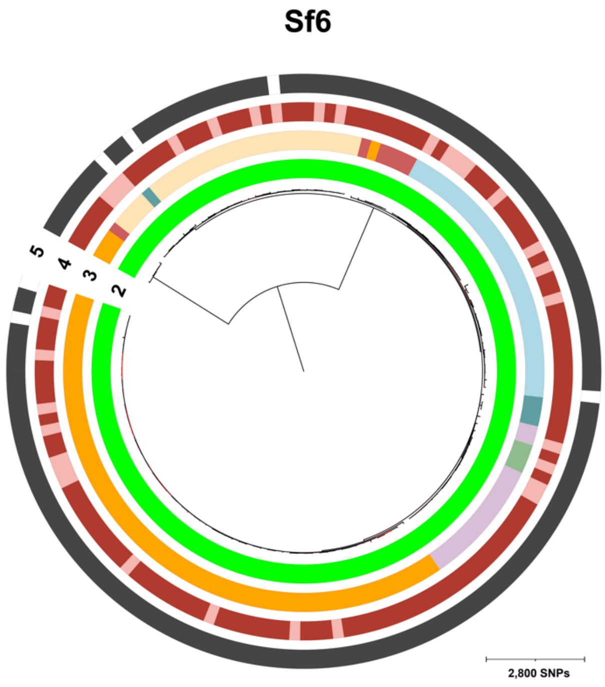

Sf6
2
3
4
5
2,800 SNPs

Supplementary Figure. 3

Phylogeny of *S. flexneri* population. ML phylogenetic trees were constructed using core genome SNPs from alignments of 659 *S. flexneri* PG1-7 genomes from GEMS with 45 publicly available genomes (A), and 147 Sf6 genomes from GEMS (B). Trees were rooted using *E. coli* genome. The outer concentric rings illustrate different genotypic and epidemiological data according to the numbered inlaid keys displayed next to the tree in A. Scale bars represents the number of SNPs.

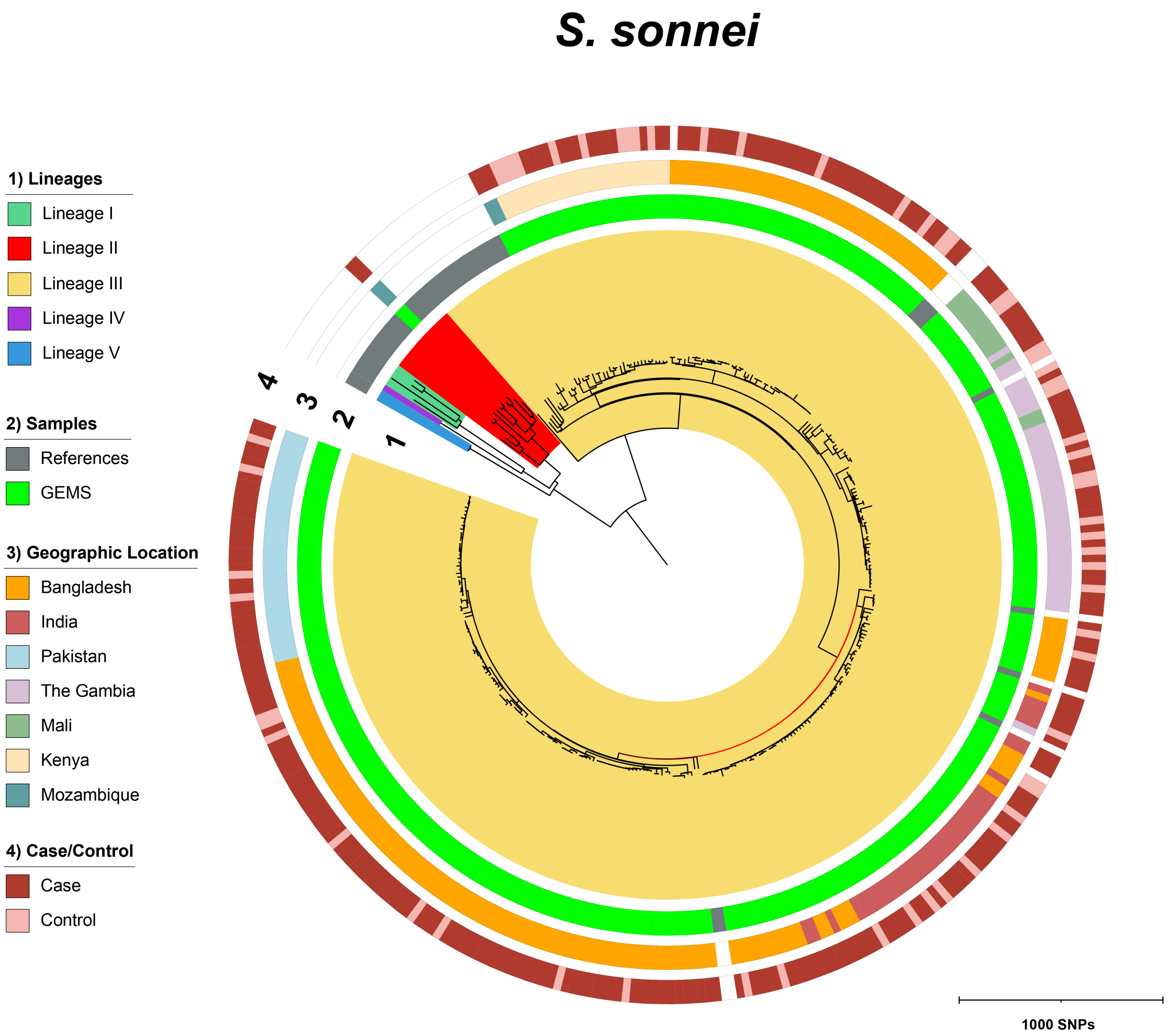

Supplementary Figure. 4

Phylogeny of *S. sonnei* population. Midpoint rooted ML phylogenetic tree constructed using core genome SNPs from alignments of 308 *S. sonnei* genomes from GEMS and 40 publicly available genomes.

A

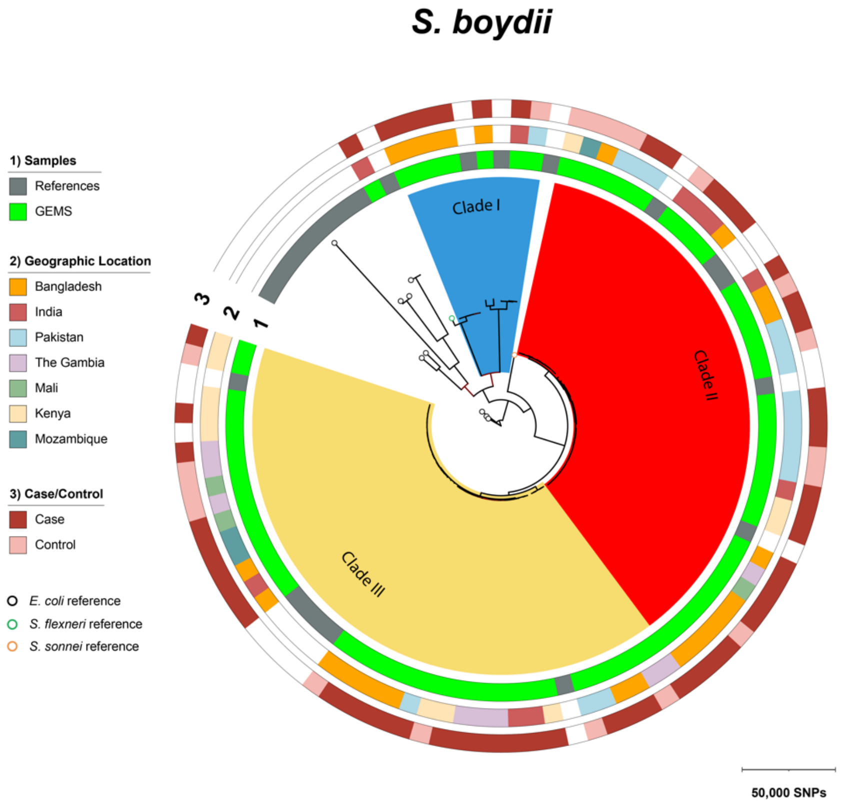
S. boydii
1) Samples
References
GEMS
2) Geographic Location
Bangladesh
India
Pakistan
The Gambia
Mali
Kenya
Mozambique
3) Case/Control
Case
Control
E. coli reference
S. flexneri reference
S. sonnei reference
Clade I
Clade II
Clade III
1
2
3
50,000 SNPs

B

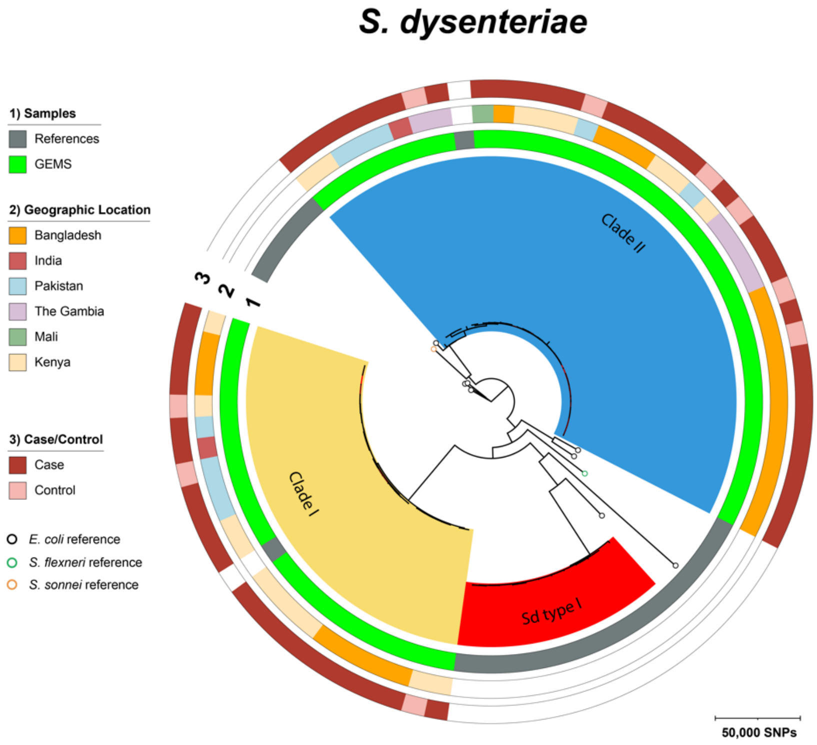
S. dysenteriae
1) Samples
References
GEMS
2) Geographic Location
Bangladesh
India
Pakistan
The Gambia
Mali
Kenya
3) Case/Control
Case
Control
E. coli reference
S. flexneri reference
S. sonnei reference
Clade I
Clade II
Sd type I
1
2
3
50,000 SNPs

Supplementary Figure. 5

Phylogeny of *S. boydii* and *S. dysenteriae* population. ML phylogenetic trees were constructed based on core genome SNPs outside region of recombination from alignments of (A) 79 *S. boydii* and (B) 60 *S. dysenteriae* genomes from GEMS and 24 publicly available genomes. Both trees were rooted using *E. coli* genome.

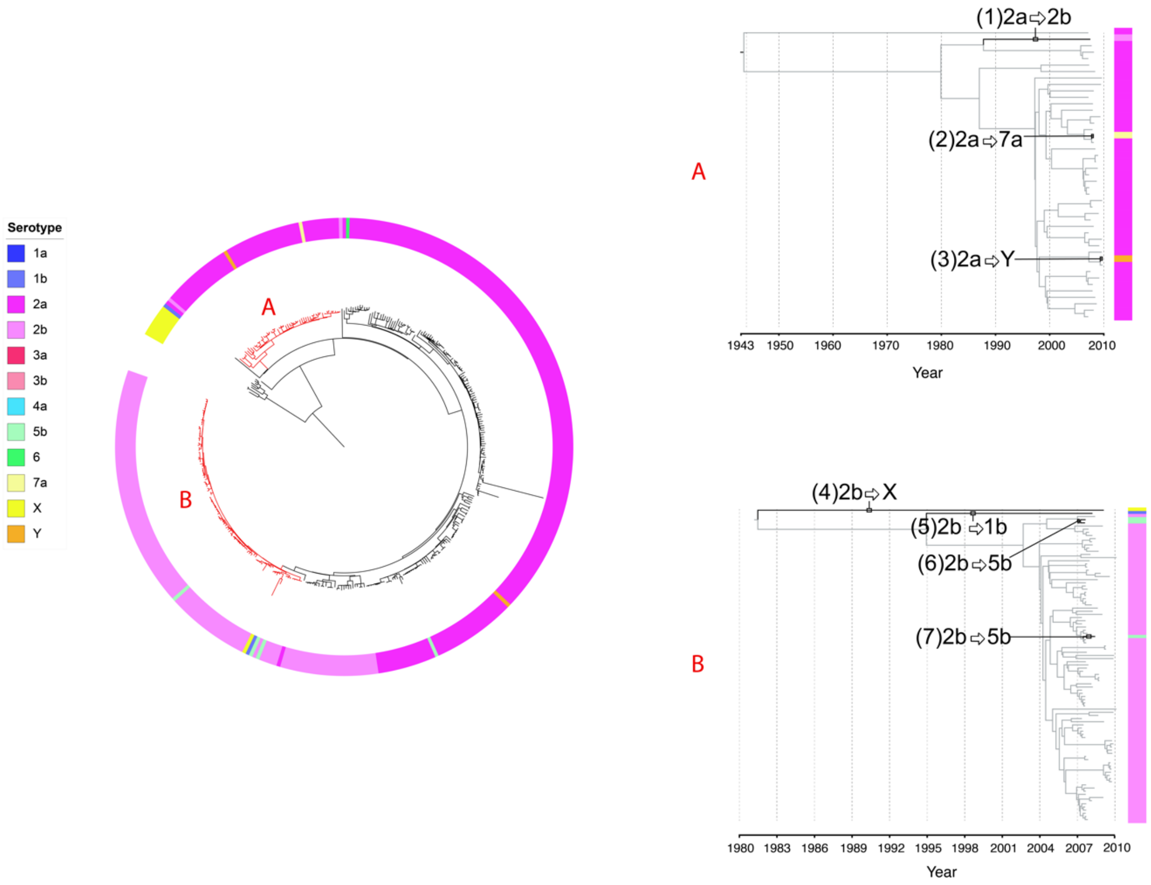

Supplementary Figure. 6

Estimation of timeframe for serotype switching among *S. flexneri* PG3 isolates. ML phylogenetic tree of *S. flexneri* PG3 (*n*=384) generated using core genome SNPs is displayed on the left, in which isolate serotype is displayed on the outer ring and coloured according to the inlaid key displayed next to the tree. The two subclades with branches highlighted in red were selected for BEAST analysis. Maximum clade credibility trees based on two subclades within PG3 are displayed on the right. Independent switching events occurring along the various phylogenetic branches are highlighted in black, labelled and annotated. BEAST estimated timeframe of divergence along the branches of the seven isolates that have undergone serotype switching are shown in table S5.

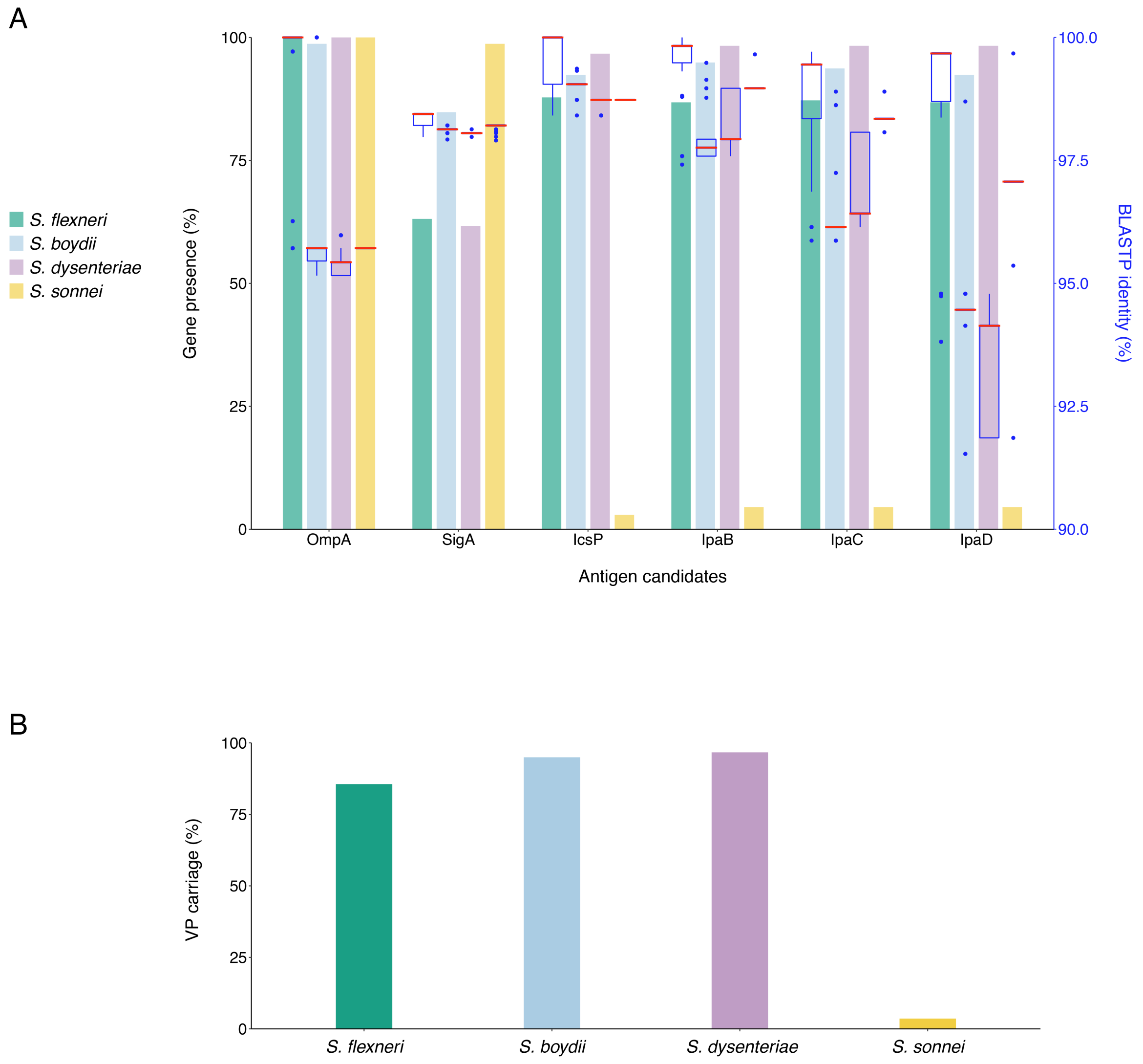

Supplementary Figure. 7

The distribution of vaccine antigen candidate and protein sequence identity among *Shigella* spp. (A) Lefthand y-axis refers to the grouped bar plot displaying presence of vaccine candidate genes identified among *Shigella* isolates from GEMS (*n*=1246). Bars are grouped by genes and coloured according to species. Righthand y-axis (blue) refers to the boxplot displaying the interquartile range, median (red) and minimum/maximum pairwise percentage identity of the amino acid sequences of antigen vaccine candidates among GEMS, compared against the reference sequences. Presence of genes were identified using BLASTn search against draft genome assemblies and amino acid sequence percentage identity were inferred using BLASTp. (B) Barplots demonstrates percentage of virulence plasmid detected among each species, as represented along the y-axis. Low percentage of virulence plasmid were detected among *S. sonnei* isolates, likely contributed by the fact that *S. sonnei* virulence plasmid is comparatively unstable and often lost during subculturing.

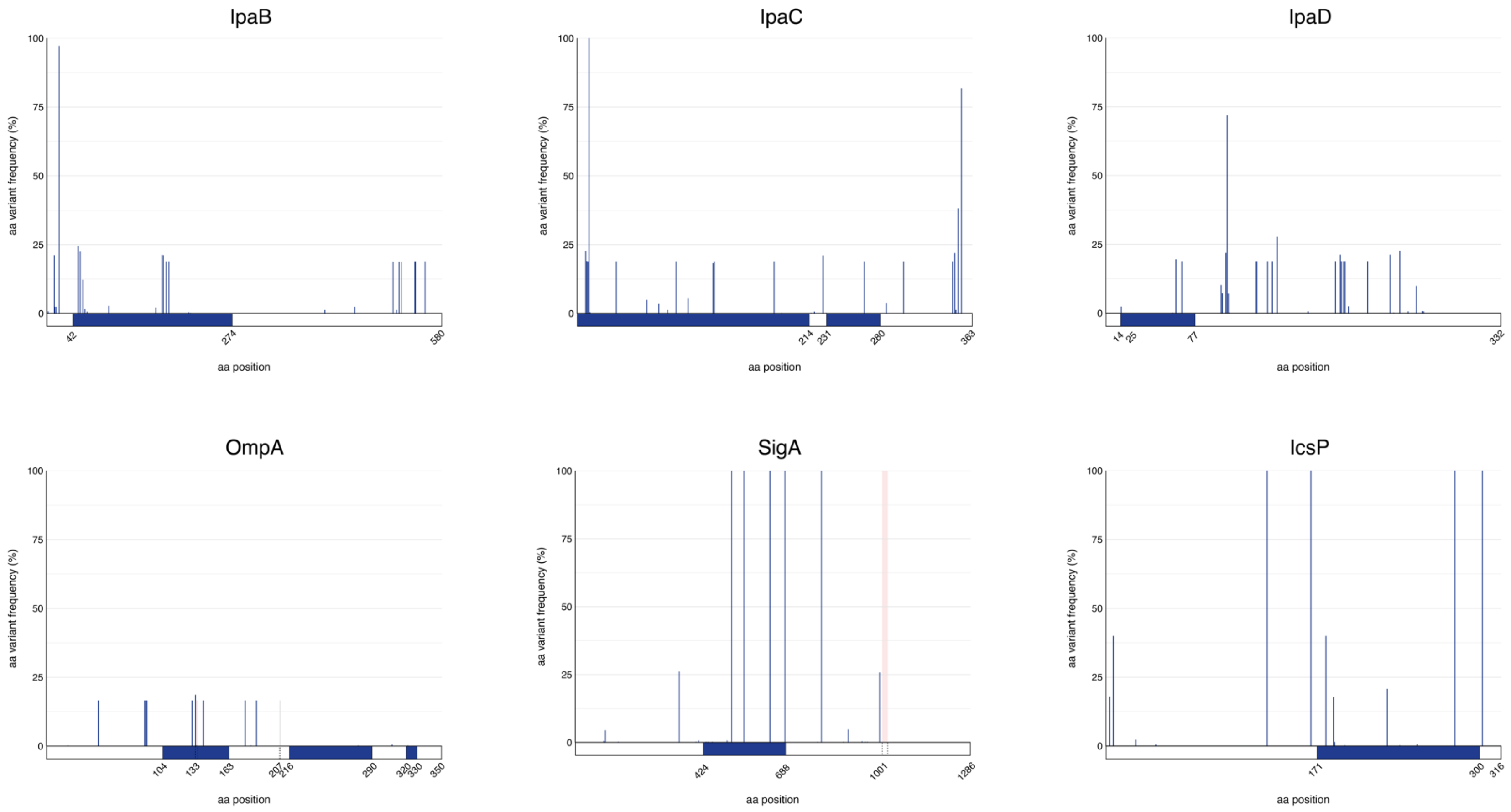

Supplementary Figure. 8

Frequency of amino acid variation among *S. flexneri* population for antigen vaccine candidates. For each protein antigen sequence, the proportion of genomes with the variant is shown along the y-axis and the position of the variant plotted along the x-axis. Grey bars highlight regions of insertions and red bars highlight deletions. Schematic of the known epitope and peptide positions (in blue) for the protein sequences are displayed below the x-axis.

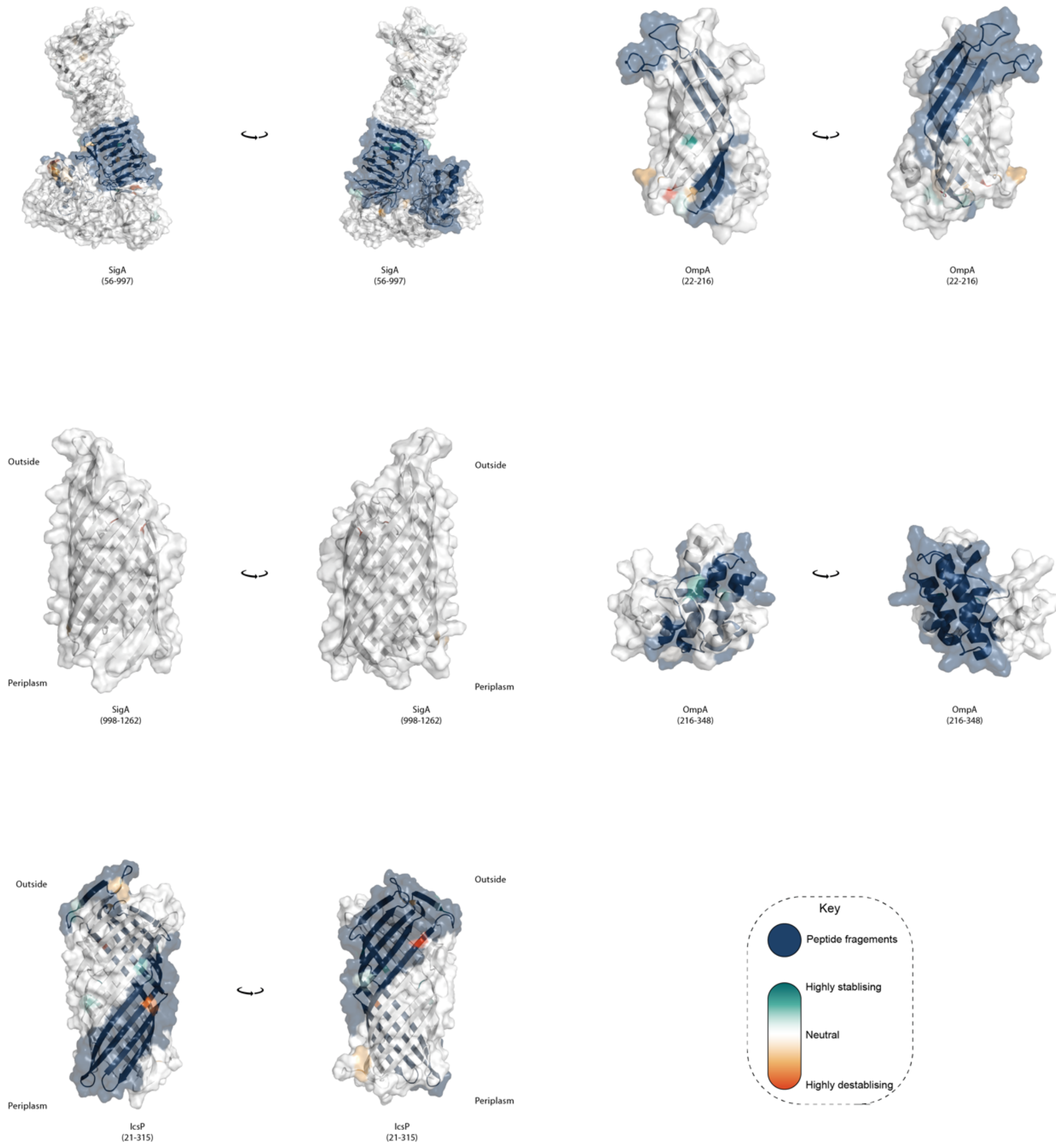

Supplementary Figure. 9

Visualization of mutations on modelled SigA, OmpA and IcsP protein antigens. Protein residues modelled are shown in brackets, peptide fragments for OmpA, SigA and IcsP that are used for vaccine development are coloured in blue. Predicted effects of mutations within the proteins are coloured using the scale shown in the key. OmpA, SigA and IcsP are orientated so that the extracellular space is located at the top of the figure, and the periplasmic space is at the bottom.

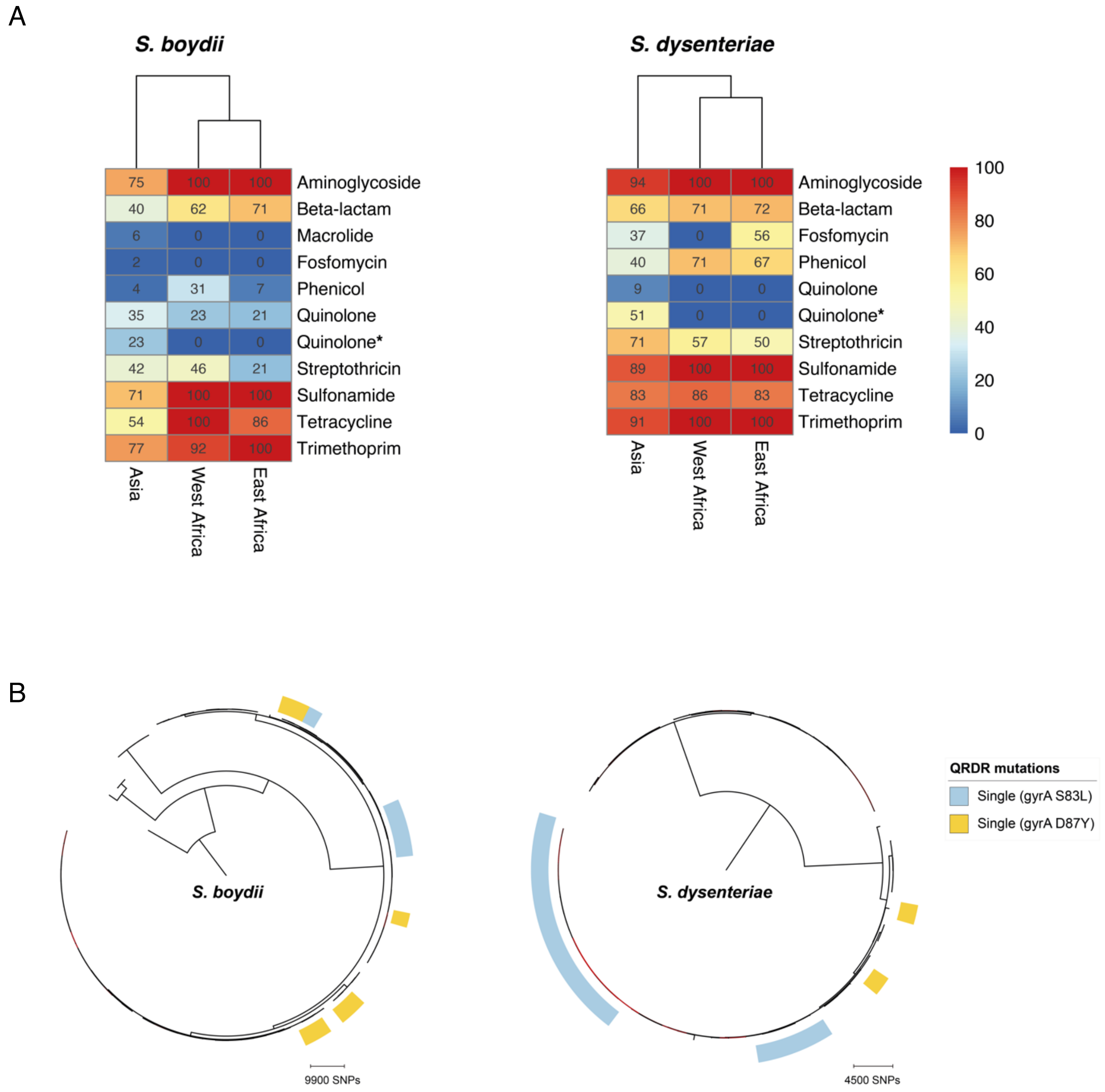

Supplementary Figure. 10

Detection of known AMR genetic determinants conferring resistance (reduced susceptibility marked with asterisk) to various drug class, grouped by region (A) and convergent evolution of ciprofloxacin resistance for *S. boydii* and *S. dysenteriae* (B).

Supplementary Table 1: *Shigella* antigen vaccine candidates examined in the current study

Vaccine candidate	Development stage	Location	Reference
IcsP (OmpP)	Preclinical	Virulence plasmid	Czerkinsky and Kim[1]
SigA	Preclinical	Chromosome (pathogenicity island)	Czerkinsky and Kim[1]
IpaB	Phase I	Virulence plasmid	Martinez-Beccera[2]; Riddle et al[3]; Tribble et al[4]
IpaC			
IpaD			
OmpA	Preclinical	Chromosome	Pore et al[5]

Supplementary Table 2: Details of *Shigella* isolates used in this study

Includes accession numbers of the sequencing reads used in the study, *Shigella* serotype, assembly statistics, year and country of isolation, condition of the child (case/control) from which the isolate was derived from as defined by GEMS, genomic subtype, AMR genes and QRDR mutations.

Please see additional file

Supplementary Table 3: Details of publicly available *E.coli/Shigella* genomes used in this study

Publicly available *E. coli/Shigella* genomes used to contextualize GEMS isolates within the established phylogroup/lineage/clade/subtype are listed in Supplementary Table 3A and *Shigella* genomes incorporated in the assessment of vaccine protein antigen variation across LMICs are listed in Supplementary Table 3B.

Please see additional file

<u>Supplementary Table 4: Association of *S. flexneri* genomic subtype / serotype with case status</u>

Genomic subtype / serotype	OR	95% CI	z statistic	*p*-value*
Sf6	0.5043	0.3198 - 0.7953	2.945	0.0032
PG1	0.5773	0.3619 - 0.9211	2.305	0.0212
PG2	0.8926	0.5102 - 1.5616	0.398	0.6906
PG3	2.3196	1.5051 - 3.5748	3.813	0.0001
PG6	1.1339	0.0582 - 22.1005	0.083	0.9339
PG7	2.9426	0.3889 - 22.2638	1.045	0.2959
1a	0.8088	0.0386 - 16.9574	0.137	0.8913
1b	0.6867	0.3942 - 1.1961	1.328	0.1843
2a	1.9329	1.1712 - 3.1900	2.578	0.0099
2b	2.2614	1.1117 - 4.5997	2.252	0.0243
3a	0.8926	0.5102 - 1.5616	0.398	0.6906
4a	0.7946	0.3230 - 1.9548	0.501	0.6167
5b	0.4798	0.0495 - 4.6540	0.633	0.5264
6	0.4829	0.3072 - 0.7590	3.155	0.0016
7a	0.6029	0.2399 - 1.5151	1.076	0.2818
Y	1.46	0.0781 - 27.3032	0.253	0.8001
X	1.6157	0.2048 - 12.7458	0.455	0.6489

*P-value was calculated using a two-sided test

Supplementary Table 5: Details of serotype determining genes facilitating *S. flexneri* (*n*=72) serotype switching

Please see additional file

Supplementary Table 6: BEAST estimated timeframe for serotype switching among *S. flexneri* PG3 isolates

Switch ID[#]	Subclade[¶]	Serotype change	Molecular serotype gene detected[&]	Median branch length (days)[$]	95% HPD branch length (days)
3	A	2a → Y	-	159	16 - 344
2	A	2a → 7a	*gtrII*	154	27 - 307
1	A	2a → 2b	*gtrII, gtrX*	7203	4792 - 10009
7	B	2b → 5b	*gtrII, gtrX*	348	244 - 479
6	B	2b → 5b	*gtrII, gtrX*	254	134 - 491
5	B	2b → 1b	*gtrI, gtrII, Oac1b*	4888	2962 - 7114
4	B	2b → X	*gtrX, gtrll*	10206	5494 - 15408

Footnotes:

[#] Serotype switching event labelled according to Extended data 6

[¶] Isolate phylogenetic subclade origin

[&] Presence of serotype determining genes, as detected by ShigaTyper, - indicates no genes were detected.

[$] Phylogenetic branch length represents divergence time, predicted by BEAST and inferred from a time-scaled tree.

Supplementary Table 7: An overview of the protein modelling

Table includes information about the antigen candidates modelled, the range of residues the proteins were modelled over, homologues used in template modelling and the QMEAN method and score.

Species	Antigen candidates	Phylogroup	Serotype	Start	Finish	Homolog	Sequence Identity	QMEAN method	Average local QMEAN score
S. flexneri	OmpA	PG3	5A	22	216	1QJP	93%	QMEANDisCo	0.46
S. flexneri	OmpA	PG3	5A	349	490	1R1M	41%	QMEANDisCo	0.31
S. flexneri	SigA	PG3	2A	56	997	3SZE	44%	QMEANDisCo	0.71
S. flexneri	SigA	PG3	2A	998	1262	2QOM	85%	QMEANDisCo	0.83
S. flexneri	IcsP	PG3	5A	21	315	1I78	60%	QMEANDisCo	0.81
S. flexneri	IpaB	PG3	5A	1	222	3U0C	76%	QMEANBrane	0.83
S. flexneri	IpaB	PG3	5A	223	553	3WXX	19%	QMEANBrane	0.79
S. flexneri	IpaC	PG3	5A	1	363	-	-	QMEANBrane	0.85
S. flexneri	IpaD	PG3	5A	1	332	3R9V	100%	QMEANBrane	0.80

Supplementary Table 8: Details of amino acid variants identified for the six antigen candidates among *S. flexneri* isolates from GEMS

Table includes variant type, variant location, reference and alternative variant, and energy score of the variant as predicted by premPS.

Please see additional file

References for Supplementary Information

1. Czerkinsky, C. & Kim, D.W. Shigella protein antigens and methods. (US Patent 8168203, 2012).
2. Martinez-Becerra, F.J. *et al.* Broadly protective Shigella vaccine based on type III secretion apparatus proteins. *Infect Immun* **80**, 1222-31 (2012).
3. Riddle, M.S. *et al.* Safety and immunogenicity of an intranasal Shigella flexneri 2a Invaplex 50 vaccine. *Vaccine* **29**, 7009-19 (2011).
4. Tribble, D. *et al.* Safety and immunogenicity of a Shigella flexneri 2a Invaplex 50 intranasal vaccine in adult volunteers. *Vaccine* **28**, 6076-85 (2010).
5. Pore, D., Mahata, N., Pal, A. & Chakrabarti, M.K. Outer membrane protein A (OmpA) of Shigella flexneri 2a, induces protective immune response in a mouse model. *PLoS One* **6**, e22663 (2011).
