# Supplementary material for: Pathogenomic analyses of Shigella isolates inform factors limiting shigellosis prevention and control across LMICs
Source: Nat Microbiol. 2022 Jan 31;7(2):251–61. doi: 10.1038/s41564-021-01054-z (PMC8813619; doi:10.1038/s41564-021-01054-z)
Supplement: Supplementary file 2 — Reporting Summary [file 41564_2021_1054_MOESM2_ESM.pdf]

nature portfolio

Corresponding author(s): Kate S. Baker

Last updated by author(s): 2021-12-03

Reporting Summary

Nature Portfolio wishes to improve the reproducibility of the work that we publish. This form provides structure for consistency and transparency in reporting. For further information on Nature Portfolio policies, see our Editorial Policies and the Editorial Policy Checklist.

Statistics

For all statistical analyses, confirm that the following items are present in the figure legend, table legend, main text, or Methods section.

n/a	Confirmed	
☐	☒	The exact sample size (*n*) for each experimental group/condition, given as a discrete number and unit of measurement
☒	☐	A statement on whether measurements were taken from distinct samples or whether the same sample was measured repeatedly
☐	☒	The statistical test(s) used AND whether they are one- or two-sided<br>*Only common tests should be described solely by name; describe more complex techniques in the Methods section.*
☐	☒	A description of all covariates tested
☒	☐	A description of any assumptions or corrections, such as tests of normality and adjustment for multiple comparisons
☐	☒	A full description of the statistical parameters including central tendency (e.g. means) or other basic estimates (e.g. regression coefficient) AND variation (e.g. standard deviation) or associated estimates of uncertainty (e.g. confidence intervals)
☐	☒	For null hypothesis testing, the test statistic (e.g. *F*, *t*, *r*) with confidence intervals, effect sizes, degrees of freedom and *P* value noted<br>*Give P values as exact values whenever suitable.*
☐	☒	For Bayesian analysis, information on the choice of priors and Markov chain Monte Carlo settings
☒	☐	For hierarchical and complex designs, identification of the appropriate level for tests and full reporting of outcomes
☒	☐	Estimates of effect sizes (e.g. Cohen's *d*, Pearson's *r*), indicating how they were calculated

Our web collection on statistics for biologists contains articles on many of the points above.

Software and code

Policy information about availability of computer code

Data collection	Microsoft Excel for Mac v16.50
Data analysis	Trimmomatic v0.38, FastQC v0.11.6, MultiQC v1.7, BWA mem v0.7.17, SAMtools v1.9-47, Picard v2.21.1-SNPSHOT, QualiMap v2.2.2, Bcftools v1.9-80, PHASTER (https://phaster.ca/), BEDTools v2.28.0, Gubbins v2.3.4, IQ-TREE v2.0-rc2, iTOL v6.1.1, snp-dists v0.7.0, TempEst v1.5.3, BEAUTI v2.6.1, BEAST2 v2.6.1, bModelTest, Tracer v1.7.1, LogCombiner v2.6.1, TreeAnnotator v2.6.0, Unicycler v0.4.7, QUAST v5.0.2, Prokka v1.13.3, Roary v3.12.0, Vegan v2.5-7, ShigaTyper v1.0.6, SRST2 v2, screen_assembly (https://github.com/shimbalama/screen_assembly), AliView v1.26, HHPred, RosettaCM source relase-188, trRosetta, QMEAN v4.2.0, QMEANbrane v4.2.0, QMEANDisCo v4.2.0, PremPS, AMRFinderPlus v3.9.3, UpSetR v2.1.3, R v4.0.3 MedCalc's odds ratio calculator v20

For manuscripts utilizing custom algorithms or software that are central to the research but not yet described in published literature, software must be made available to editors and reviewers. We strongly encourage code deposition in a community repository (e.g. GitHub). See the Nature Portfolio guidelines for submitting code & software for further information.

Data

Policy information about availability of data

All manuscripts must include a data availability statement. This statement should provide the following information, where applicable:

- Accession codes, unique identifiers, or web links for publicly available datasets
- A description of any restrictions on data availability
- For clinical datasets or third party data, please ensure that the statement adheres to our policy

Short read sequences supporting the findings of this study have been deposited in the European Nucleotide Archive (https://www.ebi.ac.uk/ena/) under the project accession number PRJEB45383. Accession numbers for isolates used in this study are listed in Supplementary Table 2. Publicly available sequences were downloaded

from GenBank (https://www.ncbi.nlm.nih.gov/genbak/), Sequence Read Archive (https://www.ncbi.nlm.nih.gov/sra), European Nucleotide Archive (https://www.ebi.ac.uk/ena) or EnteroBase (https://enterobase.warwick.ac.uk), with accession numbers listed in Supplementary Table 3. Phylogenetic trees and antigen protein models have been deposited in FigShare: (DOI:10.6084/m9.figshare.14743833).

Field-specific reporting

Please select the one below that is the best fit for your research. If you are not sure, read the appropriate sections before making your selection.

☒ Life sciences ☐ Behavioural & social sciences ☐ Ecological, evolutionary & environmental sciences

For a reference copy of the document with all sections, see nature.com/documents/nr-reporting-summary-flat.pdf

Life sciences study design

All studies must disclose on these points even when the disclosure is negative.

Sample size

Samples from this study were derived from stool samples of children from the Global Enteric Multicenter Study (GEMS).

All isolates which were confirmed to be *Shigella* through biochemical tests and agglutination with antisera were whole genome sequenced, resulting in a sample size of 1,344.

Data exclusions

Any sample which failed quality control of sequence reads were excluded. Specifically, any samples that was identified as a serotype other than Shigella following computational typing of genome sequence data and phylogenetic analysis were excluded. Samples with a mean sample depth of coverage <10x and samples with total assembly size outside the range of <4Mbp and >6.4Mbp were excluded.

Replication

Robust maximum likelihood phylogenetic trees were generated with 1000 bootstrap replicates to determine branch support. Bayesian evolutionary analysis was run. on five independent chains, each of length 250,000,000. Replicates were well supported and individual branch support is available embedded in the tree files provided in FigShare repository 10.6084/m9.figshare.14743833

Randomization

The GEMS Shigella isolates represent a systematic collection of bacteria taken during a previously described (and appropriately randomised) case-control study. Details of randomisation during the original study can be found in the methodological paper under PubMedCentral accession PMC3502307.

Blinding

Group allocations (and by extension blinding) is not relevant for this study

Reporting for specific materials, systems and methods

We require information from authors about some types of materials, experimental systems and methods used in many studies. Here, indicate whether each material, system or method listed is relevant to your study. If you are not sure if a list item applies to your research, read the appropriate section before selecting a response.

Materials & experimental systems

n/a	Involved in the study
☒	☐ Antibodies
☒	☐ Eukaryotic cell lines
☒	☐ Palaeontology and archaeology
☒	☐ Animals and other organisms
☒	☐ Human research participants
☒	☐ Clinical data
☒	☐ Dual use research of concern

Methods

n/a	Involved in the study
☒	☐ ChIP-seq
☒	☐ Flow cytometry
☒	☐ MRI-based neuroimaging
