# Supplementary material for: Pathogenomic analyses of Shigella isolates inform factors limiting shigellosis prevention and control across LMICs
Source: Nat Microbiol. 2022 Jan 31;7(2):251–61. doi: 10.1038/s41564-021-01054-z (PMC8813619; doi:10.1038/s41564-021-01054-z)
Supplement: Supplementary file 3 — Peer Review Information [file 41564_2021_1054_MOESM3_ESM.pdf]

naturereseach

Peer Review Information

Journal: Nature Microbiology
Manuscript Title: Pathogenomic analyses of Shigella isolates inform factors limiting shigellosis prevention and control across LMICs
Corresponding author name(s): Kate Baker

Reviewer Comments & Decisions:

Decision Letter, initial version:

Dear Kate,

Thank you for your patience while your manuscript "Informing shigellosis prevention and control through pathogen genomics" was under peer-review at Nature Microbiology. It has now been seen by 3 referees, whose expertise and comments you will find at the end of this email. Although they find your work of some potential interest, they have raised a number of concerns that will need to be addressed before we can consider publication of the work in Nature Microbiology.

In particular, you will see that the referees mentions the need to use more publicly available data besides GEMS (referee #1), to explain how the classification by phylogroups instead of serotypes can provide a better way of design a vaccine for Shigella (referee #1 and referee #2), to correlate the genomic diversity with phenotype or antigen variability (referee #2). Referee #3 raises questions regarding the representativeness of the samples for the conclusions drawn in terms of geographical regions and over time (are the conclusions drawn still pertinent in 2021 given the study is based on data from 2011?) and asks to expand on how often surveillance should be performed based on the results obtained.

Should further experimental data allow you to address all of these criticisms, we would be happy to look at a revised manuscript.

We are committed to providing a fair and constructive peer-review process. Please do not hesitate to contact us if there are specific requests from the reviewers that you believe are technically impossible or unlikely to yield a meaningful outcome.

We strongly support public availability of data. Please place the data used in your paper into a public data repository, if one exists, or alternatively, present the data as Source Data or Supplementary Information. If data can only be shared on request, please explain why in your Data Availability

Statement, and also in the correspondence with your editor. For some data types, deposition in a public repository is mandatory - more information on our data deposition policies and available repositories can be found at https://www.nature.com/nature-research/editorial-policies/reporting-standards#availability-of-data.

Please include a data availability statement as a separate section after Methods but before references, under the heading "Data Availability". This section should inform readers about the availability of the data used to support the conclusions of your study. This information includes accession codes to public repositories (data banks for protein, DNA or RNA sequences, microarray, proteomics data etc…), references to source data published alongside the paper, unique identifiers such as URLs to data repository entries, or data set DOIs, and any other statement about data availability. At a minimum, you should include the following statement: "The data that support the findings of this study are available from the corresponding author upon request", mentioning any restrictions on availability. If DOIs are provided, we also strongly encourage including these in the Reference list (authors, title, publisher (repository name), identifier, year). For more guidance on how to write this section please see:
http://www.nature.com/authors/policies/data/data-availability-statements-data-citations.pdf

If revising your manuscript:

* Include a "Response to referees" document detailing, point-by-point, how you addressed each referee comment. If no action was taken to address a point, you must provide a compelling argument. This response will be sent back to the referees along with the revised manuscript.

* If you have not done so already we suggest that you begin to revise your manuscript so that it conforms to our Article format instructions at http://www.nature.com/nmicrobiol/info/final-submission. Refer also to any guidelines provided in this letter.

* Include a revised version of any required reporting checklist. It will be available to referees (and, potentially, statisticians) to aid in their evaluation if the manuscript goes back for peer review. A revised checklist is essential for re-review of the paper.

When submitting the revised version of your manuscript, please pay close attention to our href="https://www.nature.com/nature-research/editorial-policies/image-integrity">Digital Image Integrity Guidelines. and to the following points below:

-- that unprocessed scans are clearly labelled and match the gels and western blots presented in figures.
-- that control panels for gels and western blots are appropriately described as loading on sample processing controls
-- all images in the paper are checked for duplication of panels and for splicing of gel lanes.

Finally, please ensure that you retain unprocessed data and metadata files after publication, ideally archiving data in perpetuity, as these may be requested during the peer review and production process or after publication if any issues arise.

Please use the link below to submit a revised paper:

{redacted}

Note: This url links to your confidential homepage and associated information about manuscripts you may have submitted or be reviewing for us. If you wish to forward this e-mail to co-authors, please delete this link to your homepage first.

Nature Microbiology is committed to improving transparency in authorship. As part of our efforts in this direction, we are now requesting that all authors identified as 'corresponding author' on published papers create and link their Open Researcher and Contributor Identifier (ORCID) with their account on the Manuscript Tracking System (MTS), prior to acceptance. This applies to primary research papers only. ORCID helps the scientific community achieve unambiguous attribution of all scholarly contributions. You can create and link your ORCID from the home page of the MTS by clicking on 'Modify my Springer Nature account'. For more information please visit please visit www.springernature.com/orcid.

If you wish to submit a suitably revised manuscript we would hope to receive it within 6 months. If you cannot send it within this time, please let us know. We will be happy to consider your revision, even if a similar study has been accepted for publication at Nature Microbiology or published elsewhere (up to a maximum of 6 months).

In the meantime we hope that you find our referees' comments helpful.

Yours sincerely,

{redacted}

****
Reviewer Expertise:

Referee #1: Shigella epidemiology and resistance in LMICs
Referee #2: Vaccines for AMR
Referee #3: Enteric pathogenomics

Reviewer Comments:

Reviewer #1 (Remarks to the Author):

Interesting paper. The authors take the Shigella GEMS isolates amd performed a genomic analysis and linked the data back to immunization policy/vaccine development and potentail treatment regimes. The output is interesting and provides a new way to integrate genomics with public health policy, whether this is an entirely novel approach is questionable, but it is a more applied way of using genomic data.

Some comments include:

1, why link to only GEMS data? What not expand to all other publically availible data sources? It seems this is a way to present the data in a unique way, but isn't really integrated into the GEMS study - apart from location.
2. The genomic data is interesting, but is there a way to develop this work to generate more funcational data around the bacteria in question. It seems that the paper generates some nice ideas and hypotheses that then need some follow up.
3. The serotype switching data is clearly important, but needs to be better integrated in the potential mechanisms for protection from vaccines. i.e. surely shigella vaccines will likley be OMV or conjugate based using an Shigella polysaccaride, so the serotype is key to this.
4. The AMR data is fairly obvious and mirrors the existing literature, the WHO guidelines have been out of date for some time, not sure what this adds to the paper.

In short the paper is nice approach to using genomic data to inform vaccine design and could utilise additional public data and needs to provide additional justification for a range of potebntail vaccine candidates.

Reviewer #2 (Remarks to the Author):

Bengtsson and coauthors have performed genomic analyses of a large number of Shigella isolates sampled during the GEMS study from 7 different countries in sub-Saharan Africa and South Asia. Results from this work are important to further understand Shigella serotypes diversity and prevalence in LMICs, serotype switching dynamics and to support the need for a multivalent cross-protective vaccine to fight Shigella. Furthermore, the study shows convergent evolution of resistance against ciprofloxacin, currently recommended by WHO, making the need for a vaccine an even higher global priority. Taken together this is the largest representative dataset of Shigella genomes from LMICs.

The manuscript is well-written, and the methodology used is solid and well described. The way in which the data are presented is very clear.

Comments and suggestions:
Genomic diversity:

• Diversity is described based on single nucleotide polymorphism (as % of SNP over total genomic base pairs).
• No phenotypic characterization or antigen structure analysis was conducted as part of this work to correlate genomic diversity with phenotype or antigen variability
• 20-25 diversity was found in flexneri vs sonnei. This is reported as a major difference that should prioritize vaccination against sonnei. In reality, this correlates with the larger number of serotypes of flexneri (14) vs sonnei (1), so diversity does not seem much different once normalized by serotype. This seems an important aspect that could be included in the interpretation of the data.
• Since there are both case and control samples involved in the study, the Authors should elaborate more on the differences that may exist between the two groups. The phylogeny of the control strains can be inferred from the trees in Figures S2, S3 and S4 but otherwise no more information is provided, and no description is included in the text with regards to cases and controls. For instance: was any difference noted in the AMR profiles of cases vs. controls? Any difference in the frequency or in the localization of mutations in the genes encoding the modelled antigens in cases vs. controls? Was the frequency of serotype switching different in cases vs. controls? Although there is clear reference to

the GEMS study, it would probably be useful to remind the reader of how cases and controls were defined.
• Considerations reported in the results should be moved to the discussion (e.g. on the value of vaccination in lines 94-96, suggested vaccination approach in 128-130, etc).
• SNP analysis identified multiple lineages for S. flexneri, phylogenic association with country of origin, comparable S.flexneri diversity across region, and varied based on region for the other species. This seems to have value to understand how pathogens transmit and clonally expand, rather than guide vaccine development (particularly if only based on SNP and not on serotype).
• Classification by genomic subtype (phylogroups) is suggested as a better way to design a vaccination strategy, rather than based on serotype. It should be explained how this could drive the design of a vaccine based on surface exposed antigens.
• No evidence of association of between serotypes and genomic diversity was identified.
• Serotype switching is infrequent, but the analysis revealed that increased selective pressure (such as from introduction of a vaccine) could selective pressure and induce serotype switching.
• The paper describes sequence variability for proteins currently considered as potential candidate recombinant protein vaccines. The analysis of such a large panel is valuable (though not adding additional evidence that any of these antigens could provide protection).
AMR
• 95% of isolates included AMR determinants against multiple antimicrobial classes
• Resistance was frequent for first line antibiotics (fluoroquinolones) but infrequent against antibiotics currently used as second line treatments. This is a useful snapshot of the current AMR situation.
• Convergent evolution was found in mutations that confer resistance to first line treatment

Minor comments:
-2 vaccines are licensed for Shigella even if with limited use to Russia and China; abstract and line 55 could state that no vaccines are widely available against Shigella
-Abstract, line 36: I would suggest expanding a bit to clarify that the approach used here for Shigella could be extended to other pathogens
-Line 62: I would add Mani at al Vaccine. 2016 Jun 3;34(26):2887-2894 as more recent reference
-Among the technology mentioned in the introduction for the development of a vaccine against Shigella I would mention also GMMA, that are proposed as delivery system for the O-antigen but where proteins and lipoproteins can be additional key elements for efficacy.
Line 226: please define what FQR is
-Paragraphs starting line 83 and 132: are the SNPs detected particularly in specific region of the genome (i.e. specific genes?), if so, can you better discuss the envisaged output on the phenotype?
-Line 200 and a general comment: can you perform or have you performed some experiments to assess the binding of antibodies to the different antigens to corroborate the in silico data (probably this is the major limitation of the study, although being already discussed suggest either to add data or better discuss this limitation).
-Conclusion: could the authors comment some more on the heterogeneity among Shigella protein antigens and provide more clear indication on the impact that these results can have on vaccine design?
-Shigella should always be capitalized.
-Figure S1: S. boydii and S. dysenteriae instead of S. boydii and S. boydii
-Figure S5: Shigella in italic

Minor points:

- Line 83: “LIMC” should probably be “LMIC”.
- Line 158: please explain what “changes in the composition of phage-encoded gtr and oac genes “ means.
- Legend to Figure S1: “S. boydi” should probably be replaced by “S. dysenteriae”.
- Figure S6: panels A and B should probably be swapped.
- Figure S8: the description in the legend does not correspond to what is shown on the right and on the left of the Figure.
- Figure S9: panel B is described in the legend but not shown in the Figure.

Reviewer #3 (Remarks to the Author):

This manuscript by Bengtsson et al. is a very well-done study using pathogen genomics to inform control of shigellosis, based on samples obtained from the Global Enteric Multicenter Study dataset from 2007-11.

My two main questions center on the representativeness of this sample for the conclusions it aims to draw. First, it would be helpful for the authors to address the extent to which the conclusions from this sample from 2007-11 remain pertinent in 2021. Is there evidence to support either the stability in the disease-causing Shigella populations or to suggest that there is population variation over the intervening 10 years?

Second, the GEMS set-up provides a sampling framework that resulted in the largest collection of Shigella samples and genomes from LMICs. However, it’s not clear to me that the seven clinical sites are representative of their regions, countries, or continents. Although GEMS is systematic, is there evidence that the samples obtained from these seven sites were representative at these scales? If not, how does that impact the authors’ conclusions?

With the idea that population genomics could inform prevention and control as the authors propose, it would be helpful if the authors could then address how often surveillance should be performed, and what surveillance strategy would be most useful to achieve goals of targeting vaccine serotypes or antigens and assessing antimicrobial susceptibility. If the goal is to show that surveillance is beneficial for management and prevention of shigellosis, then it would be helpful to address how much surveillance (how many samples, from how many areas, over what time period) would be needed to make sure you get it right, or at least not too wrong.

Minor points:
- The authors claim that the ratio of disease to overall genomic diversity points to the value of vaccination against S. sonnei. I’m curious as to why the overall genomic diversity is the relevant metric here, as opposed to the amount of disease and the extent to which there are conserved antigenic targets (which is not necessarily tied to the overall genetic diversity).
- Given the distance between Sf6 and the other S. flexneri phylogroups, I suggest that the phylogenetic tree for this species be split into one for Sf6 and one for the others to help resolve the trees.
- Since only individual sites in seven countries were sampled in GEMS, four in Africa and three in Asia

(all in the subcontinent), I suggest considering describing the geographic locations by the cities where the sampling occurred, rather than by their countries / continents.
- For the BEAST2 analysis, could the authors make available their xml and output files, perhaps in github or another file sharing site?
- Figs 1 and 2: it would be helpful to identify the GEMS sampling sites within each country on the map in Fig 1 and to clarify these are individual sites in Fig 2.
- Fig 5a: What is a 'normal' AMR genotype profile? The display is a bit confusing – what is the x-axis? It's not clear that it has any significance, so I'm confused why some of the points are clustered / overlapping. Histograms may be an easier to interpret representation of the data.

Author Rebuttal to Initial comments

Reviewer #1 (Remarks to the Author):

Interesting paper. The authors take the Shigella GEMS isolates and performed a genomic analysis and linked the data back to immunization policy/vaccine development and potential treatment regimes. The output is interesting and provides a new way to integrate genomics with public health policy, whether this is an entirely novel approach is questionable, but it is a more applied way of using genomic data.

Some comments include:

1. why link to only GEMS data? What not expand to all other publicly available data sources? It seems this is a way to present the data in a unique way, but isn't really integrated into the GEMS study - apart from location.

We would like to thank the reviewer for raising this point. The reason we chose to focus on GEMS isolates was because as a systematically collected (i.e. unbiased) modern collection of samples from regions most impacted by the disease it has the greatest relevance for informing public health, the ultimate aim of our study. While *Shigella* infection occurs worldwide, in high income countries it is often associated with foreign travel and causes only a fraction of the global disease burden. Therefore, LMICs are the main regions to target for *Shigella* vaccination and implementation of prevention and control strategies. And, while GEMS is from 2007-2011, it is the most up to date collection of representative *Shigella* genomes from LMICs. The systematic sampling of GEMS is an important feature as publicly available data often have sampling biases with regard to e.g. clinical, geographical, AMR variables (or unknown/undescribed biases). Therefore, incorporating public data *en masse* would have introduced sampling bias into

the collection, skewing important results which rely on the representativeness of the sample (e.g. the prevalence of AMR and serotype switching frequency).

However, one area of the work which would benefit from considering the greater diversity of *Shigella* from LMICs is the variation of vaccine antigens, into which we have now incorporated appropriate public data. Specifically, all publicly available (n=236) genomes sampled from LMICs between 2007 and 2021 (genomes and metadata in table S2) were also analysed. Results from this expanded analysis show that the majority (90%) of antigenic variation was captured by GEMS data, with additional variants in the public data highlighting the need for ongoing surveillance in the region. A description of the findings can be found at NL300-306 and NL314-336, and updated at fig S14, fig S15, fig S16, table S7 and methods (NL652-655). We thank the reviewer for this helpful suggestion which helped us extend the insight provided in the manuscript.

2. The genomic data is interesting, but is there a way to develop this work to generate more functional data around the bacteria in question. It seems that the paper generates some nice ideas and hypotheses that then need some follow up.

We agree with the reviewer that the data here generate important new specific directions for directing wet laboratory/applied experimental work with *Shigella*, some of which are highlighted in the manuscript (e.g. "*immunological experiments will be required to determine the true impact of these variations*", at NL340-342). However, conducting comprehensive extension functional studies across the fields of vaccine design, immunology, and antimicrobial resistance is beyond the scope of this study. It is our intention and great desire that our timely, digestible, publicly accessible, and information-rich overview of these important pathogens will accelerate intelligent experimental design across other groups working on *Shigella* and inspire those working on other pathogens to consider a similar approach.

3. The serotype switching data is clearly important, but needs to be better integrated in the potential mechanisms for protection from vaccines. i.e. surely shigella vaccines will likely be OMV or conjugate based using an Shigella polysaccaride, so the serotype is key to this.

We have further elaborated on how serotype switching may impact *Shigella* vaccination at NL251-254"*Consequently, serotype switching may impact the long-term effectiveness of vaccines that only provide serotype-specific protection against O-antigens. This highlights the advantages of protein-based or multivalent component approaches, such as the Invaplex or live attenuated vaccines that target both carbohydrates and protein antigens*"

4. The AMR data is fairly obvious and mirrors the existing literature, the WHO guidelines have been out of date for some time, not sure what this adds to the paper.

While we are grateful to the reviewer for their reflections on this section, we respectfully disagree on this point, as did Reviewer 2 who stated that "This is a useful snapshot of the current AMR situation". Although the AMR data from this study is consistent with available information, the existing literature is scarce, and a robust comparable view on AMR prevalence across LMICs is missing. The contemporaneous, systematic sampling across GEMS provides the necessary framework to robustly support our valuable, geographically comparative assertion that while WHO recommended FQR treatment may still be effective in African sites, it requires updating in the Asian region.

In short the paper is nice approach to using genomic data to inform vaccine design and could utilise additional public data and needs to provide additional justification for a range of potential vaccine candidates.

Reviewer #2 (Remarks to the Author):

Bengtsson and coauthors have performed genomic analyses of a large number of Shigella isolates sampled during the GEMS study from 7 different countries in sub-Saharan Africa and South Asia. Results from this work are important to further understand Shigella serotypes diversity and prevalence in LMICs, serotype switching dynamics and to support the need for a multivalent cross-protective vaccine to fight Shigella. Furthermore, the study shows convergent evolution of resistance against ciprofloxacin, currently recommended by WHO, making the need for a vaccine an even higher global priority. Taken together this is the largest representative dataset of Shigella genomes from LMICs.

The manuscript is well-written, and the methodology used is solid and well described. The way in which the data are presented is very clear.

Comments and suggestions:
Genomic diversity:

• Diversity is described based on single nucleotide polymorphism (as % of SNP over total genomic base pairs).

• No phenotypic characterization or antigen structure analysis was conducted as part of this work to correlate genomic diversity with phenotype or antigen variability

The reasons for this have been detailed in response to Reviewer 1's comment regarding functional work (above).

• 20-25 diversity was found in flexneri vs sonnei. This is reported as a major difference that should prioritize vaccination against sonnei. In reality, this correlates with the larger number of serotypes of flexneri (14) vs sonnei (1), so diversity does not seem much different once normalized by serotype. This seems an important aspect that could be included in the interpretation of the data.

We thank the reviewer for this comment and agree with the reviewer that taking serotype diversity into account is important for the comparison (in fact highlighting this was part of the motivation for presenting the interspecific comparison in such a strict way). However, we agree that a helpful extension of the comparison would be to look at serotype-specific data for *S. flexneri* compared with *S. sonnei*. As such, we have performed the analysis and normalised diversity by serotype which revealed that *S. sonnei* caused more disease relative to genomic diversity (albeit less pronounced) relative the five other most common serotypes of *Shigella* in GEMS. This data is presented in a new supplementary figure (fig S1) and integrated in NL121-124 "*However, when stratified by serotype/subserotype or genomic subtype, S. sonnei had a more comparable diversity and less pronounced increase in disease burden relative to genomic diversity (1.1 – 22.1-fold higher by serotype/subserotype and 1.2 – 4.9-fold higher by genomic subtype) (fig. S1 and fig. S2)*"

• Since there are both case and control samples involved in the study, the Authors should elaborate more on the differences that may exist between the two groups. The phylogeny of the

control strains can be inferred from the trees in Figures S2, S3 and S4 but otherwise no more information is provided, and no description is included in the text with regards to cases and controls. For instance: was any difference noted in the AMR profiles of cases vs. controls? Any difference in the frequency or in the localization of mutations in the genes encoding the modelled antigens in cases vs. controls? Was the frequency of serotype switching different in cases vs. controls? Although there is clear reference to the GEMS study, it would probably be useful to remind the reader of how cases and controls were defined.

We thank the reviewer for this suggestion and have provided details on how cases and controls were defined in GEMS at NL175-176 and in methods NL459-463, and the distribution of cases and controls is available in the supplementary data (new fig S5 – S7 and table S1). An in-depth exploration of the differences between case and control samples represents a significant body of work for which follow up would be integral and thus would greatly delay the release of this important data set and analyses. Indeed, we are currently conducting a thorough GWAS analysis complemented by functional work in a complementary, but distinct, follow-on study.

• Considerations reported in the results should be moved to the discussion (e.g. on the value of vaccination in lines 94-96, suggested vaccination approach in 128-130, etc).

Results and discussions were combined throughout this manuscript, but we would be happy to report results and discussions separately upon editorial advice.

• SNP analysis identified multiple lineages for S. flexneri, phylogenic association with country of origin, comparable S.flexneri diversity across region, and varied based on region for the other species. This seems to have value to understand how pathogens transmit and clonally expand, rather than guide vaccine development (particularly if only based on SNP and not on serotype).

While we agree with the reviewer that these analyses have value in understanding the transmission and expansion of *Shigella*, we feel that the genomic epidemiology of *Shigella* is comparatively well described in other works and that was not the main focus of this study. We aimed to capture and compare the extent of genomic diversity across different geographical sites to highlight the importance of regionally evaluating vaccine candidates (as a vaccine that works well in one region may not necessarily have the

same efficacy in another region – see old L113, new NL159-161). However, the reviewer makes an excellent point that we need to consider this by both genomic and serotypic delineations (as both are relevant for *Shigella* vaccines). Therefore, we have analysed the data which revealed that regional genomic diversity, and found this is also reflected in regional serotypic diversity. See NL158-159 and presented in a new supplementary figure (new fig. S9).

• Classification by genomic subtype (phylogroups) is suggested as a better way to design a vaccination strategy, rather than based on serotype. It should be explained how this could drive the design of a vaccine based on surface exposed antigens.

We have expanded the interpretation of this result in NL192-195 to address the reviewer's suggestion "*Hence, finding common surface exposed antigens that are conserved within phylogroups causing the major burden of disease may be an effective vaccine design approach that can provide greater efficacy than serotype-targeted vaccines*."

• No evidence of association of between serotypes and genomic diversity was identified.

• Serotype switching is infrequent, but the analysis revealed that increased selective pressure (such as from introduction of a vaccine) could selective pressure and induce serotype switching.

• The paper describes sequence variability for proteins currently considered as potential candidate recombinant protein vaccines. The analysis of such a large panel is valuable (though not adding additional evidence that any of these antigens could provide protection).

• 95% of isolates included AMR determinants against multiple antimicrobial classes.

• Resistance was frequent for first line antibiotics (fluoroquinolones) but infrequent against antibiotics currently used as second line treatments. This is a useful snapshot of the current AMR situation.

• Convergent evolution was found in mutations that confer resistance to first line treatment

Minor comments:

-2 vaccines are licensed for Shigella even if with limited use to Russia and China; abstract and line 55 could state that no vaccines are widely available against Shigella.

We thank the reviewer for pointing this out and have changed this in the abstract NL28 and in the introduction NL66.

-Abstract, line 36: I would suggest expanding a bit to clarify that the approach used here for Shigella could be extended to other pathogens

This has been expanded in the abstract NL36-38

-Line 62: I would add Mani at al Vaccine. 2016 Jun 3;34(26):2887-2894 as more recent reference

This has been updated at NL74

-Among the technology mentioned in the introduction for the development of a vaccine against Shigella I would mention also GMMA, that are proposed as delivery system for the O-antigen but where proteins and lipoproteins can be additional key elements for efficacy.

This has been added at NL79-81 "*Other strategies being explored include vaccines combining protein and serotype antigens, such as Generalized Modules of Membrane Antigens (GMMA), which involves use of outer membrane particles derived from genetically modified S. sonnei to elicit a stronger immune response*"

-Line 226: please define what FQR is

FQR is defined in the introduction at (old, L51) NL53

-Paragraphs starting line 83 and 132: are the SNPs detected particularly in specific region of the

genome (i.e. specific genes?), if so, can you better discuss the envisaged output on the phenotype?

SNPs were evaluated across the entire chromosome (excluding insertion sequences and phages) for each species and high areas of SNP density were detected using gubbins as part of the masking process for generating phylogenetic trees (as per methods). For *S. sonnei*, there were no regions of increased SNP density detected, while for other species there were various regions which were identified as possible recombination, likely reflecting the greater genetic diversity of the non-*sonnei* species. These details have now been added to manuscript (NL124-126, fig. S4).

-Line 200 and a general comment: can you perform or have you performed some experiments to assess the binding of antibodies to the different antigens to corroborate the in silico data (probably this is the major limitation of the study, although being already discussed suggest either to add data or better discuss this limitation).

We thank the reviewer for highlighting the value of our variability analysis of vaccine antigens (above), and we have now explicitly noted the limitations of our *in silico* analyses in the manuscript at NL340-342 "*However, any in silico approaches have limitations and functional immunological experiments will be required to determine the true impact of these variations on the antigen structure and its antigenicity*".

-Conclusion: could the authors comment some more on the heterogeneity among Shigella protein antigens and provide more clear indication on the impact that these results can have on vaccine design?

This has been expanded in the conclusion at NL434-436.

-Shigella should always be capitalized.

This has been altered throughout the manuscript

-Figure S1: S. boydii and S. dysenteriae instead of S. boydii and S. boydii

This has been changed

-Figure S5: Shigella in italic

This has been changed

Minor points:

- Line 83: “LIMC” should probably be “LMIC”.

This has been changed

- Line 158: please explain what “changes in the composition of phage-encoded gtr and oac genes “ means.

We apologize for the lack of clarity and have altered this sentence at NL236 “*facilitated by changes in the presence or absence of various phage-encoded*”

- Legend to Figure S1: “S. boydi” should probably be replaced by “S. dysenteriae”.

This has been changed

- Figure S6: panels A and B should probably be swapped.

This has been altered

- Figure S8: the description in the legend does not correspond to what is shown on the right and on the left of the Figure.

This has been altered

- Figure S9: panel B is described in the legend but not shown in the Figure.

We thank the reviewer for spotting this and panel B has now been added to the supplementary figure (new, fig. S13).

Reviewer #3 (Remarks to the Author):

This manuscript by Bengtsson et al. is a very well-done study using pathogen genomics to inform control of shigellosis, based on samples obtained from the Global Enteric Multicenter Study dataset from 2007-11.

My two main questions center on the representativeness of this sample for the conclusions it aims to draw. First, it would be helpful for the authors to address the extent to which the conclusions from this sample from 2007-11 remain pertinent in 2021. Is there evidence to support either the stability in the disease-causing Shigella populations or to suggest that there is population variation over the intervening 10 years?

Second, the GEMS set-up provides a sampling framework that resulted in the largest collection of Shigella samples and genomes from LMICs. However, it's not clear to me that the seven clinical sites are representative of their regions, countries, or continents. Although GEMS is systematic, is there evidence that the samples obtained from these seven sites were representative at these scales? If not, how does that impact the authors' conclusions?

We thank the reviewer for these important questions regarding the representativeness of the GEMS isolates (and thus the relevance of or findings) across time (i.e. post 2011) and space (i.e. across broader geographical areas), which we have addressed collectively below.

It is worth stating up front that the patchy surveillance and delays to securing funding, sequencing, and analysing genomes of bacterial pathogens from LMIC continues to cause unfortunate delays in insight from these critical regions. However, we have gathered encouraging evidence to support that our analysis of the GEMS isolates has far reaching and continued relevance for managing shigellosis.

Regarding the stability of *Shigella* populations across time, the Vaccine Impact on Diarrhea in Africa (VIDA) study was a 36-month long replicate of GEMS conducted between 2015 and 2018 across African sites. Laboratory analysis of VIDA has revealed a similar regional distribution of *Shigella* serotypes and prevalence of AMR[1]. Furthermore, a recent largescale genomic analysis of all publicly available *S. sonnei* revealed isolates from six South Asian countries (Bangladesh, Bhutan, India, Nepal, Pakistan and Tibet) sampled from 2012 to 2014 (and one sample from 2019) belonged to clade 3.6[2], of consistent with the majority (>80%) GEMS Asia in GEMS also belonging to this clade (table S1). These points have been addressed in NL162-171.

Furthermore, in our response to reviews, we have expanded the *S. flexneri* analysis to include all (n=236) publicly available genomes collected between 2007-2021 across LMICs, including regions not represented in GEMS such as East Asia and South Africa. This revealed that these isolates were phylogenetically admixed with GEMS isolates (new, fig S14) and added only 15 (of 148) further amino acid variations for consideration in our study, indicating the need for ongoing surveillance (discussed at NL305-306 and NL346-349).

It is also worth noting that the health centres performing surveillance during GEMS (ranging from 1 to 11 per country) were selected with the aim of being representative based on a spectrum of child health indicators, such as variations in the prevalence of malaria, HIV and etc, across rural, urban and periurban settings[3,4]. Thus, providing a broad view of enteric disease epidemiology from each region. Please also see the response to Reviewer 3's comment (below) regarding the representativeness of GEMS locations.

Collectively, this evidence supports GEMS providing a valuable and relevant overview of shigellosis in these important and under sampled areas.

With the idea that population genomics could inform prevention and control as the authors propose, it would be helpful if the authors could then address how often surveillance should be performed, and what surveillance strategy would be most useful to achieve goals of targeting vaccine serotypes or antigens and assessing antimicrobial susceptibility. If the goal is to show that surveillance is beneficial for management and prevention of shigellosis, then it would be helpful to address how much surveillance (how many samples, from how many areas, over what time period) would be needed to make sure you get it right, or at least not too wrong.

We thank the reviewer for this suggestion and agree that it is an important point to address. Although the development of a mature, statistically inferred surveillance strategy is outside the scope of this study, we have provided important parameters (such as the estimate of serotype switching time frames and prevalence) for those working directly in public health. As our temporal analysis estimated serotype switching to occur within an average of 348 days we suggest that annual surveillance is appropriate for monitoring for serotype replacement following introduction of a vaccination programme and we highlight that WGS is a superior strategy for surveillance compared with serotypic methods for *Shigella*. This has been added in the conclusion at NL438–443 "*Taken together, this study demonstrates the benefit of genomics in guiding prevention and control of shigellosis, providing further impetus to continue working to overcoming the challenges associated with the implementation of WGS for pathogen surveillance in LMICs. Finally, our results suggest that annual Shigella surveillance would likely identify serotype switching, which would be especially important following the introduction of a vaccination programme*".

Minor points:

- The authors claim that the ratio of disease to overall genomic diversity points to the value of vaccination against S. sonnei. I'm curious as to why the overall genomic diversity is the relevant metric here, as opposed to the amount of disease and the extent to which there are conserved antigenic targets (which is not necessarily tied to the overall genetic diversity).

The majority of the antigen vaccine targets (IpaB, IpaC, IpaD and IcsP) currently in development are encoded on the virulence plasmid, which is unstable in *S. sonnei* and

missing in majority (>90%) of the *S. sonnei* isolates from GEMS. Thus, it was not possible to measure the diversity of those antigen targets in relation to disease burden. However, there is good phylogenetic congruence between *Shigella* phylogroups and virulence plasmids [ref Connor et al 2015] hence we used the overall genomic diversity as a proxy measure the ratio of disease burden to diversity. Furthermore, it may be that some final targets of vaccination are not yet described in the literature.

- Given the distance between Sf6 and the other S. flexneri phylogroups, I suggest that the phylogenetic tree for this species be split into one for Sf6 and one for the others to help resolve the trees.

We have changed fig S2 (new, fig S5) so that phylogenetic tree for Sf6 and PG1- 7 isolates are separate.

- Since only individual sites in seven countries were sampled in GEMS, four in Africa and three in Asia (all in the subcontinent), I suggest considering describing the geographic locations by the cities where the sampling occurred, rather than by their countries / continents.

We thank the reviewer for this warning of extrapolation of our findings. In addition to addressing Reviewer 2's comments regarding representativeness (above), we have also now included the city names of the seven countries from GEMS in Figure 1A. For the reasons outlined above and in line with other published studies on GEMS[3-9], we have described the geographic locations by their country names.

- For the BEAST2 analysis, could the authors make available their xml and output files, perhaps in github or another file sharing site?

We have uploaded the xml and BEAST output files onto FigShare (DOI: 10.6084/m9.figshare.14743833).

- Figs 1 and 2: it would be helpful to identify the GEMS sampling sites within each country on the map in Fig 1 and to clarify these are individual sites in Fig 2.

For reasons already described above, and as our analysis did not include data on city or medical centre, providing this level of granularity on the sampling is inappropriate, and that the information is extensively described elsewhere[3,4].

- Fig 5a: What is a 'normal' AMR genotype profile? The display is a bit confusing – what is the x-axis? It's not clear that it has any significance, so I'm confused why some of the points are clustered / overlapping. Histograms may be an easier to interpret representation of the data.

We thank the reviewer for pointing out this lack of clarity and helpful suggestion. We have changed the Figure 5a to a histogram instead (new, Fig 5) and agree it is more digestible in this format.

Decision Letter, first revision:

Dear Kate,

Thank you for submitting your revised manuscript "Informing shigellosis prevention and control through pathogen genomics" (NMICROBIOL-21061500A). It has now been seen by the original referees and their comments are below. The reviewers find that the paper has improved in revision, and therefore we'll be happy in principle to publish it in Nature Microbiology, pending minor revisions to satisfy the referees' final requests and to comply with our editorial and formatting guidelines.

If the current version of your manuscript is in a PDF format, please email us a copy of the file in an editable format (Microsoft Word or LaTex)-- we can not proceed with PDFs at this stage.

We are now performing detailed checks on your paper and will send you a checklist detailing our editorial and formatting requirements in about a week. Please do not upload the final materials and make any revisions until you receive this additional information from us.

Thank you again for your interest in Nature Microbiology Please do not hesitate to contact me if you have any questions.

Sincerely,

{redacted}

Reviewer #1 (Remarks to the Author):

Most of the identified issues within the text have been resolved, it is a nice manuscript and well presented piece of work. I am not wholly convinced by some of the argumentes presented, but i do see the value of exploiting genomic data to inform vaccine and therapeutic approaches, especially for Shigella. I am not sure if there is enormous novelty or the work represents a major paradigm shift without bolting on an enormous amount of laboratory work. But as a genomics paper the work is

excellent and the approach places a more applied spin on the analysis, which makes it an interesting read with some well backed up opinions. I enjoyed reading it and like the dive into translation. Nice paper.

Reviewer #2 (Remarks to the Author):

The authors have answered the queries of this reviewer. The manuscript is suitable for publication in the present form.

Reviewer #3 (Remarks to the Author):

The authors have reasonable responses to each of my points. The work is exciting and important, and there will be much of interest to follow up on.

Decision Letter, final checks:

Dear Dr. Baker,

Thank you for your patience as we've prepared the guidelines for final submission of your Nature Microbiology manuscript, "Informing shigellosis prevention and control through pathogen genomics" (NMICROBIOL-21061500A). Please carefully follow the step-by-step instructions provided in the attached file (an additional file will be sent in a separate email), and add a response in each row of the table to indicate the changes that you have made. Please also check and comment on any additional marked-up edits we have proposed within the text. Ensuring that each point is addressed will help to ensure that your revised manuscript can be swiftly handed over to our production team.

We would like to start working on your revised paper, with all of the requested files and forms, as soon as possible (preferably within two weeks). Please get in contact with us if you anticipate delays.

When you upload your final materials, please include a point-by-point response to any remaining reviewer comments.

If you have not done so already, please alert us to any related manuscripts from your group that are under consideration or in press at other journals, or are being written up for submission to other journals (see: https://www.nature.com/nature-research/editorial-policies/plagiarism#policy-on-duplicate-publication for details).

In recognition of the time and expertise our reviewers provide to Nature Microbiology's editorial process, we would like to formally acknowledge their contribution to the external peer review of your

manuscript entitled "Informing shigellosis prevention and control through pathogen genomics". For those reviewers who give their assent, we will be publishing their names alongside the published article.

Nature Microbiology offers a Transparent Peer Review option for new original research manuscripts submitted after December 1st, 2019. As part of this initiative, we encourage our authors to support increased transparency into the peer review process by agreeing to have the reviewer comments, author rebuttal letters, and editorial decision letters published as a Supplementary item. When you submit your final files please clearly state in your cover letter whether or not you would like to participate in this initiative. Please note that failure to state your preference will result in delays in accepting your manuscript for publication.

Cover suggestions

As you prepare your final files we encourage you to consider whether you have any images or illustrations that may be appropriate for use on the cover of Nature Microbiology.

Covers should be both aesthetically appealing and scientifically relevant, and should be supplied at the best quality available. Due to the prominence of these images, we do not generally select images featuring faces, children, text, graphs, schematic drawings, or collages on our covers.

We accept TIFF, JPEG, PNG or PSD file formats (a layered PSD file would be ideal), and the image should be at least 300ppi resolution (preferably 600-1200 ppi), in CMYK colour mode.

If your image is selected, we may also use it on the journal website as a banner image, and may need to make artistic alterations to fit our journal style.

Please submit your suggestions, clearly labeled, along with your final files. We'll be in touch if more information is needed.

Nature Microbiology has now transitioned to a unified Rights Collection system which will allow our Author Services team to quickly and easily collect the rights and permissions required to publish your work. Approximately 10 days after your paper is formally accepted, you will receive an email in providing you with a link to complete the grant of rights. If your paper is eligible for Open Access, our Author Services team will also be in touch regarding any additional information that may be required to arrange payment for your article.

Please note that you will not receive your proofs until the publishing agreement has been received through our system.

Please note that <i>Nature Microbiology</i> is a Transformative Journal (TJ). Authors may publish their research with us through the traditional subscription access route or make their paper immediately open access through payment of an article-processing charge (APC). Authors will not be required to make a final decision about access to their article until it has been accepted. Find out more about Transformative Journals

<B>Authors may need to take specific actions to achieve compliance with funder and institutional open access mandates. For submissions from January 2021, if your research is supported by a funder that requires immediate open access (e.g. according to Plan S principles) then you should select the gold OA route, and we will direct you to the compliant route where possible. For authors selecting the subscription publication route our standard licensing terms will need to be accepted, including our self-archiving policies. Those standard licensing terms will supersede any other terms that the author or any third party may assert apply to any version of the manuscript.

For information regarding our different publishing models please see our Transformative Journals page. If you have any questions about costs, Open Access requirements, or our legal forms, please contact ASJournals@springernature.com.

Please use the following link for uploading these materials:
{redacted}

If you have any further questions, please feel free to contact me.

Best regards,

{redacted}

Reviewer #1:
Remarks to the Author:
Most of the identified issues within the text have been resolved, it is a nice manuscript and well presented piece of work. I am not wholly convinced by some of the argumentes presented, but i do see the value of exploiting genomic data to inform vaccine and therapeutic approaches, especially for Shigella. I am not sure if there is enormous novelty or the work represents a major paradigm shift without bolting on an enormous amount of laboratory work. But as a genomics paper the work is excellent and the approach places a more applied spin on the analysis, which makes it an interesting read with some well backed up opinions. I enjoyed reading it and like the dive into translation. Nice paper.

Reviewer #2:
Remarks to the Author:
The authors have answered the queries of this reviewer. The manuscript is suitable for publication in the present form.

Reviewer #3:
Remarks to the Author:
The authors have reasonable responses to each of my points. The work is exciting and important, and there will be much of interest to follow up on.

Final Decision Letter:

Dear Kate,

I am pleased to accept your Article "Pathogenomic analyses of Shigella isolates inform factors limiting shigellosis prevention and control across LMICs" for publication in Nature Microbiology. Thank you for having chosen to submit your work to us and many congratulations.

Over the next few weeks, your paper will be copyedited to ensure that it conforms to Nature Microbiology style. We look particularly carefully at the titles of all papers to ensure that they are relatively brief and understandable.

Once your paper is typeset, you will receive an email with a link to choose the appropriate publishing options for your paper and our Author Services team will be in touch regarding any additional information that may be required. Once your paper has been scheduled for online publication, the Nature press office will be in touch to confirm the details.

After the grant of rights is completed, you will receive a link to your electronic proof via email with a request to make any corrections within 48 hours. If, when you receive your proof, you cannot meet this deadline, please inform us at rjsproduction@springernature.com immediately. You will not receive your proofs until the publishing agreement has been received through our system

Due to the importance of these deadlines, we ask you please us know now whether you will be difficult to contact over the next month. If this is the case, we ask you provide us with the contact information (email, phone and fax) of someone who will be able to check the proofs on your behalf, and who will be available to address any last-minute problems.

Acceptance of your manuscript is conditional on all authors' agreement with our publication policies (see https://www.nature.com/nmicrobiol/editorial-policies). In particular your manuscript must not be published elsewhere and there must be no announcement of the work to any media outlet until the publication date (the day on which it is uploaded onto our website).

Please note that <i>Nature Microbiology</i> is a Transformative Journal (TJ). Authors may publish their research with us through the traditional subscription access route or make their paper immediately open access through payment of an article-processing charge (APC). Authors will not be required to make a final decision about access to their article until it has been accepted. Find out more about Transformative Journals

<B>Authors may need to take specific actions to achieve <a

href="https://www.springernature.com/gp/open-research/funding/policy-compliance-faqs"> compliance with funder and institutional open access mandates. For submissions from January 2021, if your research is supported by a funder that requires immediate open access (e.g. according to Plan S principles) then you should select the gold OA route, and we will direct you to the compliant route where possible. For authors selecting the subscription publication route our standard licensing terms will need to be accepted, including our self-archiving policies. Those standard licensing terms will supersede any other terms that the author or any third party may assert apply to any version of the manuscript.

If you have any questions about our publishing options, costs, Open Access requirements, or our legal forms, please contact ASJournals@springernature.com

An online order form for reprints of your paper is available at https://www.nature.com/reprints/author-reprints.html. All co-authors, authors' institutions and authors' funding agencies can order reprints using the form appropriate to their geographical region.

We welcome the submission of potential cover material (including a short caption of around 40 words) related to your manuscript; suggestions should be sent to Nature Microbiology as electronic files (the image should be 300 dpi at 210 x 297 mm in either TIFF or JPEG format). Please note that such pictures should be selected more for their aesthetic appeal than for their scientific content, and that colour images work better than black and white or grayscale images. Please do not try to design a cover with the Nature Microbiology logo etc., and please do not submit composites of images related to your work. I am sure you will understand that we cannot make any promise as to whether any of your suggestions might be selected for the cover of the journal.

You can now use a single sign-on for all your accounts, view the status of all your manuscript submissions and reviews, access usage statistics for your published articles and download a record of your refereeing activity for the Nature journals.

To assist our authors in disseminating their research to the broader community, our SharedIt initiative provides you with a unique shareable link that will allow anyone (with or without a subscription) to read the published article. Recipients of the link with a subscription will also be able to download and print the PDF.

As soon as your article is published, you will receive an automated email with your shareable link.
